# Design, Synthesis, and Biological Evaluation of an Allosteric Inhibitor of HSET that Targets Cancer Cells with Supernumerary Centrosomes

**DOI:** 10.1016/j.chembiol.2013.09.012

**Published:** 2013-11-21

**Authors:** Ciorsdaidh A. Watts, Frances M. Richards, Andreas Bender, Peter J. Bond, Oliver Korb, Oliver Kern, Michelle Riddick, Paul Owen, Rebecca M. Myers, Jordan Raff, Fanni Gergely, Duncan I. Jodrell, Steven V. Ley

**Affiliations:** 1 Department of Chemistry, University of Cambridge, Lensfield Road, Cambridge CB2 1EW, UK; 2 Cancer Research UK Cambridge Institute, University of Cambridge, Li Ka Shing Centre, Robinson Way, Cambridge CB2 ORE, UK; 3 Unilever Centre for Molecular Science Informatics, Department of Chemistry, University of Cambridge, Lensfield Road, Cambridge 1EW, UK; 4 Cambridge Crystallographic Data Centre, 12 Union Road, Cambridge CB2 1EZ, UK; 5 Cancer Research Technology, Ltd., Babraham Research Campus, Cambridge CB2 3AT, UK; 6 Cancer Research Technology, Ltd., Wolfson Institute of Biomedical Research, The Cruciform Building, London WC1E 6BT, UK; 7 Sir William Dunn School of Pathology, University of Oxford, South Parks Road, Oxford OX1 3RE, UK

## Abstract

Centrosomes associate with spindle poles; thus, the presence of two centrosomes promotes bipolar spindle assembly in normal cells. Cancer cells often contain supernumerary centrosomes, and to avoid multipolar mitosis and cell death, these are clustered into two poles by the microtubule motor protein HSET. We report the discovery of an allosteric inhibitor of HSET, CW069, which we designed using a methodology on an interface of chemistry and biology. Using this approach, we explored millions of compounds in silico and utilized convergent syntheses. Only compound CW069 showed marked activity against HSET in vitro. The inhibitor induced multipolar mitoses only in cells containing supernumerary centrosomes. CW069 therefore constitutes a valuable tool for probing HSET function and, by reducing the growth of cells containing supernumerary centrosomes, paves the way for new cancer therapeutics.

## Highlights

• Hybrid approach to compound discovery using chemogenomics, chemistry, and biology
• Design of an allosteric HSET inhibitor, CW069, a synthetic compound
• CW069 selectively inhibits HSET activity both in vitro and in cells
• CW069 induces multipolar anaphases by blocking centrosome clustering

## Teaser

Inhibition of HSET provides a unique opportunity to selectively target malignant cells with supernumerary centrosomes. Watts et al. develop an allosteric HSET inhibitor that does not disrupt bipolar division in normal cells but leads to multipolar mitoses in cancer cells with extra centrosomes, causing apoptosis.

## Introduction

DNA replication, followed by equal chromosome segregation, ensures the accurate transmission of the genetic information to daughter cells (Hall et al., 2003; Nigg, 2002; Zyss and Gergely, 2009). In most normal and malignant cells, centrosomes act as the dominant sites for spindle pole formation (Meunier and Vernos, 2012). Centrosome duplication is also tightly controlled and occurs simultaneously with DNA replication, thereby ensuring the generation of two functional centrosomes that form the poles of the mitotic spindle (Sharp et al., 2000). In the assembly of a functional mitotic spindle, microtubule (MT) motor proteins play a central role (Cai et al., 2010; Ganem and Compton, 2004). One such protein, HSET (encoded by *KIFC1* in humans and *Kifc5a* in mice), a minus-end MT motor, is of interest in cancer due to its impact on cell division (Cai et al., 2010; Goshima et al., 2005), and the discovery of a small-molecule inhibitor of HSET forms the focus of this study.

In recent years, the importance of centrosomes, and in particular HSET, for bipolar spindle formation has attracted much attention, although the precise role of HSET in this process remains a topic for debate (Mahoney et al., 2006; Tillement et al., 2009). Recent reports have linked centrosome amplification and high HSET expression to chromosome missegregation and aneuploidy, which are hallmarks of human cancer (Marx et al., 2009). Centrosome amplification disrupts asymmetric cell division in neuroblastoma cells and causes tumorigenesis in a fly model (Basto et al., 2008), and supernumerary centrosomes are also found in most solid tumor types, forming markers for aggressiveness in breast, brain, prostate, cervix, kidney, and bladder cancers (Chan, 2011). Hence, it is increasingly apparent that supernumerary centrosomes are not only indicative of malignancy but may also drive malignant transformation (Ogden et al., 2013). However, not all cells with centrosome amplification undergo multipolar mitosis, and a key mechanism by which cells with extra centrosomes achieve a pseudo-bipolar spindle is centrosome clustering (Basto et al., 2008; Ganem et al., 2009). Although centrosome clustering prevents multipolar mitosis and cell death, it prolongs mitosis and increases the frequency of chromosome missegregation as a result of merotelic kinetochore attachments (Ganem et al., 2009; Kwon et al., 2008; Yang et al., 2008). Based on previous studies, centrosome clustering may prove to be the Achilles heel of cancer cells with supernumerary centrosomes (Basto et al., 2008), and a growing body of evidence suggests that inhibition of centrosome clustering could provide a new therapeutic strategy for tumors with a high incidence of centrosome amplification (Jordan and Wilson, 2004; Ogden et al., 2012). Accordingly, in this work, we hypothesized that inhibition of centrosome clustering could provide an alternative approach to cancer treatment.

A key protein that is known to be crucial for centrosome clustering is HSET (Ncd in flies) (Basto et al., 2008; Kwon et al., 2008). This protein is a member of the Kinesin 14 family of MT motor proteins, which are force-generating enzymes that facilitate movement along MTs within the cell (Mountain et al., 1999). Although the precise role of HSET in cell division is not clear, previous evidence suggests that it is essential for the survival of cancer, but not normal, cells (Ganem et al., 2009; Kwon et al., 2008). High HSET expression levels are strongly correlated with metastasis of non-small cell lung cancer to the brain, pointing to an association between HSET, centrosome amplification, and tumorigenesis (Cai et al., 2010; Gordon et al., 2001; Grinberg-Rashi et al., 2009). Knockdown of HSET in normal retinal pigment epithelial 1 (RPE-1) cells or the breast cancer cell line MCF-7 (which does not have a high incidence of centrosome amplification) does not inhibit bipolar spindle formation, and cells undergo normal division (Kleylein-Sohn et al., 2012; Kwon et al., 2008). In contrast, knockdown of HSET in the supernumerary centrosome-containing breast cancer and neuroblastoma cell lines MDA-MB-231 and N1E-115, respectively, prevents centrosome clustering and induces cell death by multipolar anaphases (Kwon et al., 2008). Hence, the above findings point to HSET as a target of interest in cancer treatment (Basto et al., 2008; Kraljevic Pavelic et al., 2011; Krämer et al., 2011; Kwon et al., 2008).

Our aim was to develop a selective allosteric inhibitor of HSET. Therefore, using chemogenomics-based compound selection followed by hit exploration, we designed, synthesized, and biologically evaluated an inhibitor of HSET, CW069. Modeling supports binding of this inhibitor in the loop 5 cleft of the HSET motor domain, rationalizing its bioactivity. Importantly, we show that CW069 differentially affects the viability of cancer cells with supernumerary centrosomes compared with normal cells. This probe molecule will allow further investigation into the role of HSET in spindle formation, mitosis, and cancer, and provides a starting point for future drug-development efforts.

## Results

### An In Silico Model for HSET Binding Informs Compound Design and Synthesis of the Allosteric HSET Inhibitor CW069

**Figure 1:**
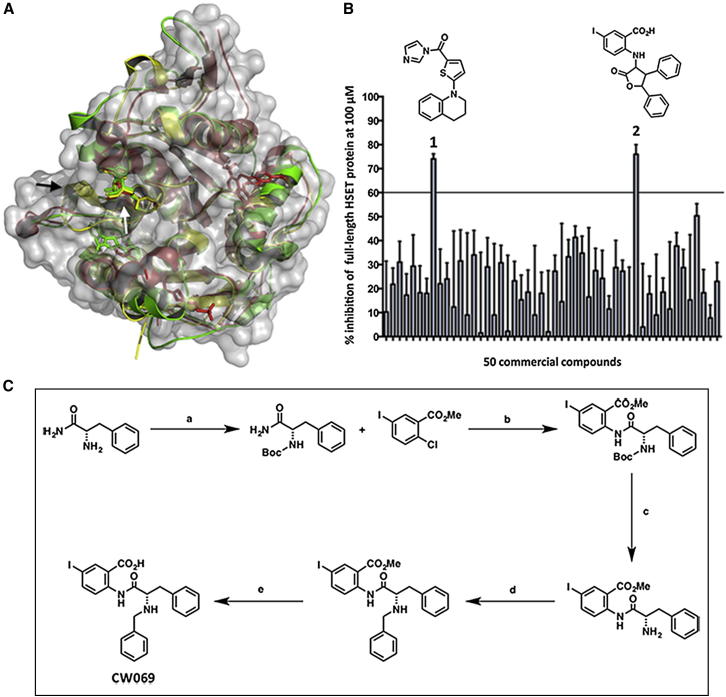
Computational Modeling Identifies Putative HSET Inhibitors In Silico (A) Overlaid motor domains of human HSET (2REP in yellow) and KSP (3ZCW in red, 2FKY in green). The figure demonstrates the smaller loop 5 of HSET (black arrow) and the potential for selective HSET binding in the loop 5 cleft (white arrow). (B) Fifty compounds were selected in silico and tested in the enzymatic assay against full-length, N-terminal, 6His-tagged human HSET (n = 3). Data are represented as mean ± SD. Two compounds showed activity greater than the 60% cutoff. (C) Improved synthetic route used to prepare the HSET inhibitor CW069. Reagents and conditions: (a) Boc_2_O, NEt_3_, CH_2_Cl_2_, RT, 3 hr, 99%; (b) 1 equiv. Pd_2_(dba)_3_, 1 equiv. Xantphos, Cs_2_CO_3_, dioxane, 110°C, 20 hr, 90%; (c) TFA, CH_2_Cl_2_, RT, 1 hr, quant.; (d) (i) NEt_3_, THF, (ii) benzaldehyde, THF, (iii) NaCNBH_3_, THF, RT, 16 hr, 81%; (e) LiOH, THF, water, 60°C, 16 hr, 85%. See also Figure S1.

Amino acid sequence alignments for the motor domains of HSET (Protein Data Bank [PDB] 2REP) and the closely related kinesin, KSP (PDB 2FKY and 3ZCW), revealed an 80% sequence similarity between the proteins in the motor domains (HSET residues 310–661). The similarity decreases to 56% in the loop 5 binding cleft alone (residues 422–432; Figure 1A). Given the high degree of similarity between the proteins, and the potential for selective binding in the loop 5 cleft, the chemogenomics principle that similar proteins bind similar ligands was applied to HSET and KSP, and an in silico model for HSET binding was developed based on nearly 500 existing inhibitors of KSP in the ChEMBL database (see Experimental Procedures for details; Klabunde, 2007). Using the model parameters, we selected 200 compounds (in silico) from 20 million possibilities and further triaged them into 50 ligands that scored highest in the bioactivity model. We acquired and tested these ligands for in vitro activity using an ADP-Glo enzymatic assay (see Experimental Procedures for details; Figure 1B).

**Figure 2:**
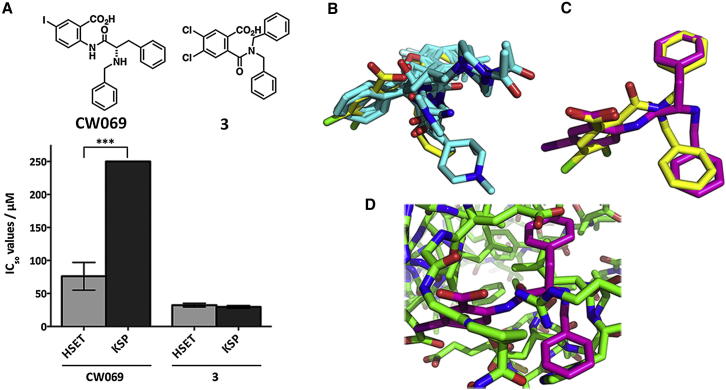
CW069 Showed Selectivity for HSET over KSP In Vitro (A) IC_50_ values for CW069 and 3 against full-length, N-terminal, 6His-tagged HSET and KSP. Compounds were tested at increasing concentrations up to 250 μM (n = 3). Data are represented as mean ± SD. Whereas compound 3 does not possess selectivity for HSET, CW069 exhibits ∼4-fold selectivity between these related proteins. (B) Overlay of compound 3 (yellow) onto seven known KSP ligands. The figure shows KSP ligands binding to the allosteric pockets of PDB 2FL2, 2FL6, 2PG2, 2Q2Y, 2Q2Z, 2UMY, and 3CJO (cyan). (C) Alignment of CW069 (magenta) onto compound 3 (yellow) in the HSET loop 5 cleft. Compound conformations are very similar. (D) Alignment of CW069 (magenta) onto the HSET loop 5 (green). As can be seen, interaction between the Arg521, Gly423, and Leu517 backbone and the carboxylic acid and amine motifs of CW069 contribute to the binding of CW069 to HSET. See also Figure S2.

From the first-pass enzymatic assay, two structures (compounds 1 and 2; Figure 1B) displayed activity, reducing HSET ATPase function by more than the (arbitrary) triage value of 60% at 100 μM. Given previously available bioactivity information for the scaffold (Neres et al., 2008), we identified compound 1 as a nonselective inhibitor of HSET. Therefore, compound 2, a commercially available ϒ-lactone benzoic acid, provided the starting point for compound design and convergent syntheses of 27 analogs (Figure S1A available online). Structure activity relationship (SAR) data were obtained against both HSET and KSP, along with 37 further commercially available compounds (Figure S1B). Of these, only our synthesized compound CW069 (Figures 1C and S1C) showed marked and selective activity against HSET in vitro. The IC_50_ value of CW069 was 75 ± 20 μM for HSET and the inhibitor showed statistically significant selectivity over KSP to the limit of the experiment (Figures 2A and S2; p < 0.001). Unlike some larger kinesin inhibitors with molecular weights greater than 500 Da, CW069 has acceptable “drug likeness” according to “Lipinski’s rule of five” (Lipinski et al., 2001) and criteria recently outlined in a study by Bickerton et al. (2012) using data from more than 700 approved drugs.

The commercially available analog 3 was equipotent against HSET and KSP, with IC_50_ values of 30 ± 4 μM and 29 ± 2 μM, respectively (Figure 2A). None of the other tested compounds inhibited HSET, indicating the steep SAR of the binding pocket.

**Figure 3:**
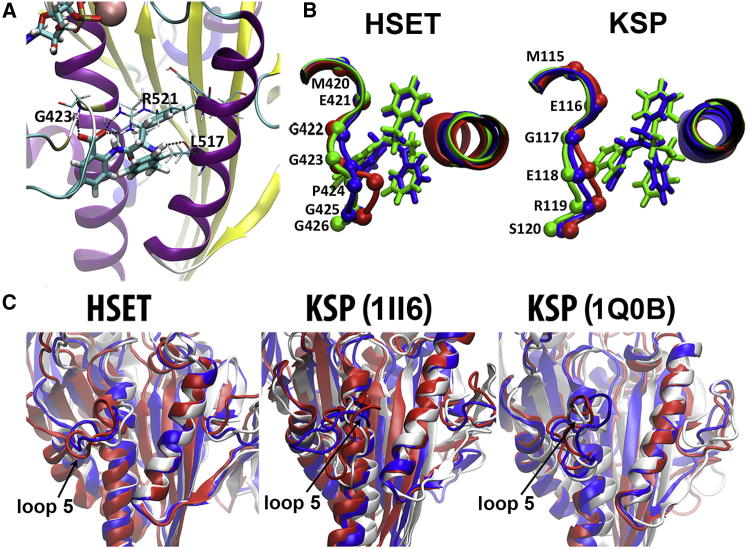
Computational Energy Minimization and Relaxation of HSET and KSP (A) Energy-minimized, relaxed HSET protein with ligand CW069 (cyan) overlaid. Predicted protein-ligand hydrogen-bond interactions between G423, R531, and L517 and carboxylate/amine groups of CW069 (dashed lines) that confer selectivity for HSET are shown. Mg^2+^ ion (pink) and ADP (top left) are present. The selectivity of CW069 for HSET is mainly driven by electrostatic interactions (see main text for details). (B) Relaxation of loop 5 in HSET and KSP during extensive energy minimization. The binding site is shown in cartoon format, with loop 5 residues labeled, for the X-ray structure (red) and the final, energy-minimized structures in the presence of CW069 (green) or compound 3 (blue). (C) Conformational changes in the binding pockets of ligand-free HSET and KSP during explicitly solvated molecular dynamics. The motor domains of HSET and KSP are shown in cartoon format, with the X-ray structure (white) overlaid on the first (red) and second (blue) most frequently observed conformations during 1 μs molecular-dynamics sampling generated for each protein. For clarity, the solvent is not shown. Clustering analysis was performed using GROMACS and VMD. See also Figure S3.

In order to generate secure binding-mode hypotheses for CW069, we used a two-step, ligand-based alignment approach. Compound 3 was first aligned onto a set of known allosteric KSP compounds and then CW069 was aligned to the superimposed conformation of compound 3. This resulted in the alignments presented in Figures 2B and 2C. As expected from a ligand-based binding-mode prediction, overlap of the ligand with protein residues was observed (Figure 2D) and further energy minimization was required (see Supplemental Experimental Procedures for details). In the overlaid, optimized structures (using CW069 and the unbound PDB structures 2REP and 1ll6), the protein-ligand interaction enthalpic energies for HSET and KSP were found to be −107 kcal/mol and −65 kcal/mol, respectively, thus supporting the experimentally observed selectivity of CW069 for HSET. A comparable degree of burial of CW069 within the hydrophobic pocket of each protein was observed and the energetic differences recorded were hence electrostatic in origin, arising from the highly favorable hydrogen bond formed between the guanidinium group of Arg521 in HSET and the carboxylate of CW069 (Figure 3A). The Arg521 side chain may also form a cation-π interaction with the exposed phenyl group of CW069 (Figure 3A). In addition, there were hydrogen-bond interactions between the respective backbone amide and carbonyl groups of Gly423 and Leu517 and the carboxylate and amine groups of CW069 (Figure 3A). Although similar interactions were possible for KSP, the absence of the key Arg521 interaction (the equivalent KSP residue is Ala218) appears to be critical for the observed selectivity.

The model also predicts that the enantiomer of CW069 binds more weakly to HSET, with a protein-ligand interaction enthalpic energy of −97 kcal/mol. This is due to less optimal interactions between Arg521 in HSET and the carboxylate of the ligand. Additionally, the enantiomer of CW069 cannot form hydrogen-bond interactions with the carbonyl groups of Gly423 and Leu517 (Figure S3).

When compound selectivity and promiscuity were rationalized against HSET and KSP, CW069 and compound 3 were found to share key structural motifs, and hence protein information was needed to further explain the experimentally observed data. Energy minimization necessitated significant relaxation of loop 5 around the binding site. For CW069, this involved cavity opening by ∼2–3 Å, whereas smaller opening or closing motions of <1 Å were observed for compound 3, explaining its lack of selectivity for HSET (Figure 3B). To explore this further while incorporating realistic protein dynamics and solvent effects, we generated 1 μs all-atom molecular-dynamics simulation ensembles for each ligand-free protein system (see Supplemental Experimental Procedures for details). In KSP, loop 5 consistently exhibited partial closure of the cavity space that would be required for binding CW069 (Figure 3C). In contrast, loop 5 of HSET was extremely dynamic around a poly-Gly motif that is absent in KSP, resulting in opening of the binding pocket and enabling accommodation of CW069 (Figure 3C). Thus, a dynamic conformational selection mechanism appears to rationalize the ability of CW069 to bind in an allosteric manner to HSET, but not KSP. Hence, by utilizing the structures of well-characterized allosteric inhibitors of KSP, we were able to rationally design an allosteric inhibitor of HSET.

### CW069 Increases Multipolar Spindles in N1E-115 Cells with Supernumerary Centrosomes without Altering Bipolar Spindle Morphology in Normal Human Dermal Fibroblast Cells

**Figure 4:**
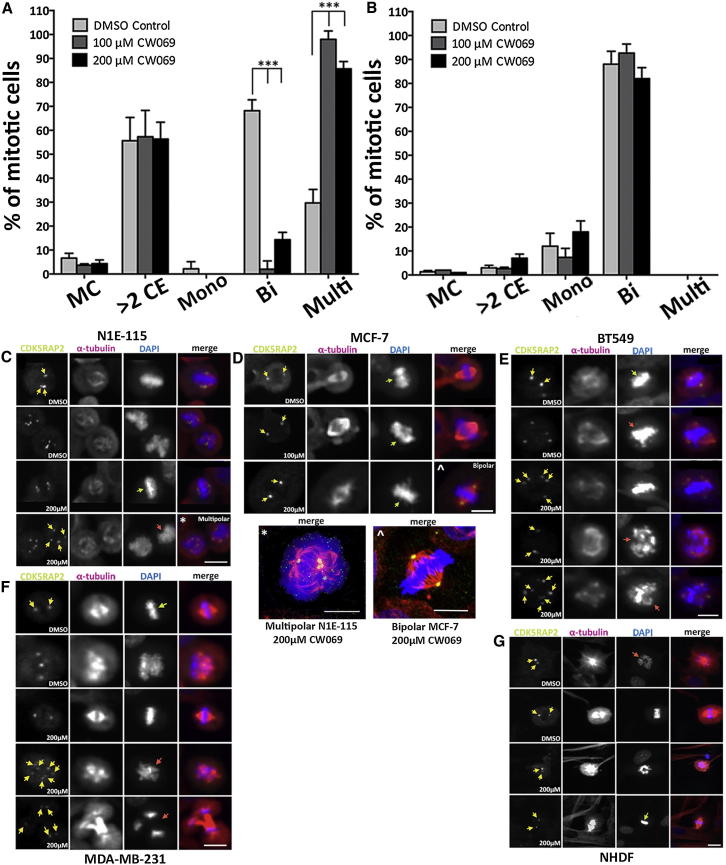
Effect of CW069 on Mitotic Spindle Morphology in Cells with Normal or Supernumerary Centrosomes (A and B) Data for N1E-115 cells (A) and NHDF cells (B). Cells were treated with DMSO control (0.2%) or 100 μM and 200 μM CW069 for 2.5 hr. Antibodies against α-tubulin, CDK5RAP2, and DAPI were employed to visualize MTs, centrosomes, and DNA, respectively. Data are represented as mean ± SD; n = 3 wells (at least 200 mitotic cells from each well were scored). Bi, bipolar; CE, centrosomes; MC, mitotic cells; Mono, monopolar; Multi, multipolar. (A) CW069 treatment increases the number of multipolar spindles and unclustered centrosomes in N1E-115 cells at 100 μM and 200 μM compound concentrations. (B) CW069 treatment does not disrupt the mitotic spindle morphology of NHDF cells, even at 200 μM compound concentration. (C–G) Representative images for (C) N1E-115 cells (bar = 25 μm), (D) MCF-7 cells (bar = 20 μm), (E) BT549 cells (bar = 20 μm), (F) MDA-MB-231 cells (bar = 25 μm), and (G) NHDF cells (bar = 25 μm). Cells were treated with DMSO control (0.2%) or 100 μM and 200 μM CW069 for 2.5 hr (n > 100). Red, MTs (anti-α-tubulin antibody and anti-mouse Alexa Fluor 647); green, centrosomes (anti-CDK5RAP2 antibody and anti-rabbit Alexa Fluor 488); blue, DNA (DAPI). Yellow arrows indicate centrosome location, green arrows indicate bipolar spindles, and red arrows indicate abnormal spindles (multipolar). (C) Examples of bipolar and multipolar mitotic spindles are shown in untreated and treated N1E-115 cells. The frequency of multipolar spindles increased when cells were treated with CW069. See also Figure S4.

In order to investigate the effect of CW069-induced HSET inhibition on mitotic spindle morphology, as previously described for HSET depletion by siRNA (Kwon et al., 2008), we first confirmed the mitotic index, centrosome numbers, and extent of mitotic multipolarity in exponentially growing cultures of untreated N1E-115 and normal human dermal fibroblast (NHDF) cells using immunofluorescence (IF). The untreated N1E-115 cancer cells displayed a high mitotic index (nearly 10%) and very high centrosome amplification (55%), and multipolar spindles in mitosis (30%; Figure 4A). In contrast, the NHDF cells showed no multipolar spindles in mitosis (0%) and a low mitotic index (1%) and centrosome amplification (3%; Figure 4B). The same data were gathered for MDA-MB-231 (Figure S4A), BT549 (Figure S4B), and MCF-7 (Figure S4C) breast cancer cells.

Using IF, we next examined the mitotic phenotypes induced by CW069 in N1E-115 and NHDF cells, with a strong focus on centrosome numbers and mitotic spindle morphology. Antibodies against α-tubulin, CDK5RAP2, and DAPI were employed to visualize MTs, centrosomes, and DNA, respectively. We hypothesized that small-molecule inhibition of HSET would give rise to mitotic spindle defects similar to those reported after HSET knockdown by siRNA transfection (Kwon et al., 2008), and this was indeed the case. Upon treatment with CW069, a significant increase in multipolar spindle formation was observed in N1E-115 cells (control 30%, 100 μM 98%, 200 μM 86%, p < 0.001; Figure 4A), but not in NHDF cells (Figure 4B). After treatment, the N1E-115 cells formed multipolar spindles due to a lack of centrosome clustering, consistent with HSET inhibition in cells with supernumerary centrosomes (Figure 4C). The mitotic spindle morphology of treated NHDF cells was unchanged compared with control (Figure 4G).

Figures 4D–4F show IF data for MDA-MB-231, BT549, and MCF-7 breast cancer cells, respectively. CW069 induced a small but significant increase in multipolar spindles in MDA-MB-231, consistent with the intermediate level of supernumerary centrosomes in the untreated cells. BT549 cells also showed increased multipolar spindles after CW069 treatment. However, CW069 did not perturb bipolar spindle formation in MCF-7 cells, in agreement with the phenotype described in a previous study using siRNA knockdown of HSET (Kwon et al., 2008), lending further validity to the mode of action of CW069 described here.

### Time-Lapse Microscopy Confirms that CW069 Recapitulates the Multipolar Anaphases and Cell Death Induced in N1E-115 Cells by Transfection with HSET siRNA

**Figure 5:**
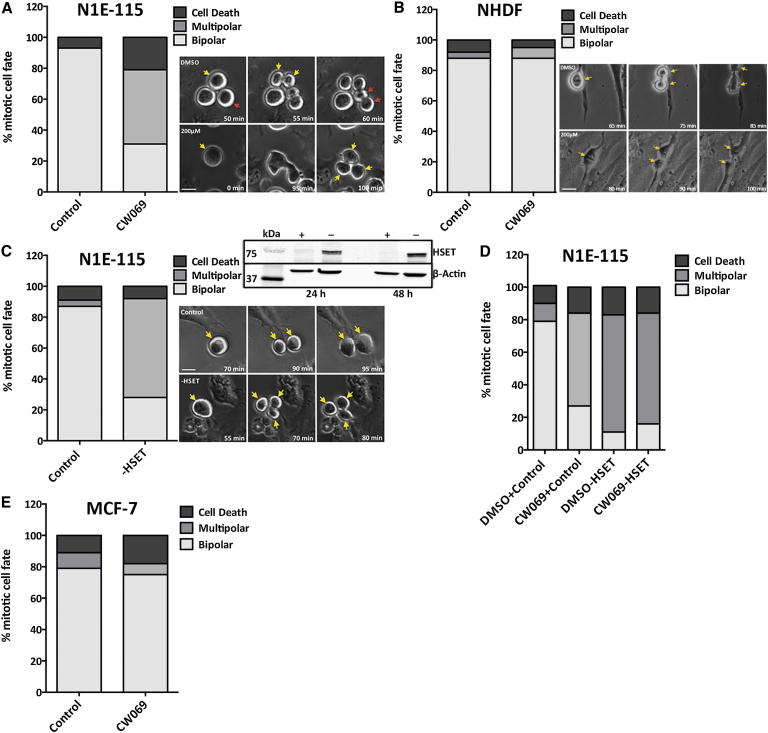
Time-Lapse Image Analysis of the Effect of CW069 on Mitotic Fate (A) N1E-115 cells treated with DMSO control (0.2%) or 200 μM CW069. Untreated cells divided normally, in a bipolar manner. CW069-treated cells divided aberrantly, with multipolar cytokinesis. (B) NHDF cells treated with DMSO control (0.2%) or 200 μM CW069. CW069-treated cells divided in the same bipolar manner as control cells. (C) N1E-115 cells transfected with control siRNA or HSET siRNA. Control cells divided in a normal bipolar manner, whereas HSET siRNA-transfected cells underwent aberrant division, with multipolar cytokinesis. (D) CW069/HSET siRNA-treated cells divided with the same increased multipolarity as observed for CW069-treated or HSET siRNA-transfected cells alone, indicating that CW069 selectively inhibits HSET activity. (E) MCF-7 cells treated with CW069 divided in the same bipolar manner as control cells (see also Figure S6). Representative examples for cell divisions are shown. The fate of at least 100 mitotic cells was scored from images taken over 1,440 min of imaging (bar = 20 μm). Yellow and red arrows denote cells directly before and after anaphase. See also Figure S5 and Movies S1 and S2.

In a previous study using time-lapse microscopy, Kwon et al. (2008) showed that siRNA knockdown of HSET induces a dramatic increase in multipolar anaphases in cancer cells with supernumerary centrosomes (N1E-115 and MDA-MB-231). Therefore, we interrogated cell division in N1E-115 cells using time-lapse imaging for further validation of target engagement. Treatment with 200 μM CW069 did indeed induce multipolar anaphase formation in N1E-115 cells (48%; Figure 5A) compared with the DMSO control (0%; Figure 5A). This result recapitulated (although to a slightly lesser extent) the effect, reported here and by others (Kwon et al., 2008), of HSET siRNA transfection (64% multipolar anaphases; Figures 5C and S5) compared with control siRNA (4% multipolar anaphases; Figure 5C). After initial aberrant divisions induced by CW069, the majority of daughter cells underwent apoptosis. This is consistent with a previous report that catastrophic aneuploidy associated with multipolar divisions prompted cell death (Kwon et al., 2008). In order to corroborate selective inhibition of HSET by CW069 in cells, we treated HSET siRNA-depleted cells with 200 μM CW069 and compared them with DMSO/control siRNA-, DMSO/HSET siRNA-, and CW069/control siRNA-treated cells (Figure 5D). In contrast to results obtained with DMSO/control siRNA (11% multipolar anaphases), the phenotypes induced by CW069/HSET siRNA, DMSO/HSET siRNA, and CW069/control siRNA were comparable (68%, 72%, and 57% multipolar anaphases, respectively). These data demonstrate that CW069 cannot further increase multipolar anaphases when HSET is absent, and thus indicate that the inhibitor is selective for HSET.

No similar increase in multipolar anaphase formation was observed upon treatment of NHDF cells with 200 μM CW069 (Figure 5B). The bipolar division of MCF-7 cells (with normal centrosome numbers) also remained unaffected by treatment with 200 μM CW069 (Figures 5E, S6A, and S6B). This too is in agreement with the hypothesis that HSET motor activity is essential for proper spindle formation in cells with centrosome amplification, but is dispensable in cells with normal centrosome numbers.

### CW069 Does Not Cause Mitotic Delay or Arrest and Suppresses the Mitotic Phenotype Induced by Inhibition of KSP in Cells

**Figure 6:**
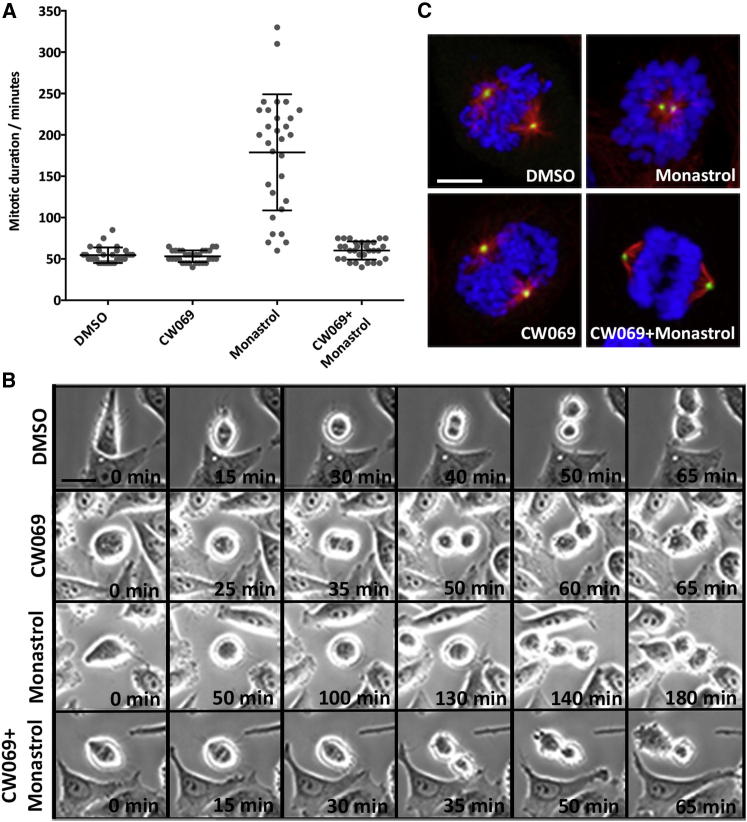
CW069 Does Not Cause Mitotic Arrest in HeLa Cells, and Antagonizes Inhibition of KSP by Monastrol (A and B) Length of MD for 0.2% DMSO-treated, 200 μM CW069-treated, and 100 μM monastrol-treated HeLa cells, and HeLa cells treated with CW069+monastrol. Time-lapse video microscopy was carried out after 60 min of treatment, every 5 min for a further 360 min. Thirty cells were scored for each treatment (bar = 8 μm). CW069 does not change the mitotic kinetics of cells and suppresses mitotic arrest induced by KSP inhibition. (C) Representative images of mitotic cells with the treatments. Red, MTs (anti-α-tubulin antibody and anti-mouse Alexa Fluor 647); green, centrosomes (anti-CDK5RAP2 antibody and anti-rabbit Alexa Fluor 488); blue, DNA (DAPI); bar = 3 μm. Data are represented as mean ± SD. Monastrol inhibition of KSP produced monopolar spindles. Proper bipolar spindle formation was largely rescued by cotreatment of CW069 and monastrol. See also Movies S3, S4, S5, and S6.

HSET is known to antagonize the activity of the related kinesin, KSP, during spindle formation (Mountain et al., 1999). Therefore, we used time-lapse video microscopy to investigate whether CW069-treated cells transit mitosis with normal kinetics, and whether the characteristic mitotic arrest induced by inhibition of KSP (Mayer et al., 1999) could be suppressed by cotreatment with CW069. To this end, HeLa cells were treated with either DMSO control (0.2%), 200 μM CW069, 100 μM monastrol, or CW069+monastrol for 60 min before being imaged every 5 min for a further 360 min. Mitotic duration (MD) was defined as the period of time between when the cells began to round up and the onset of anaphase. No statistical difference was recorded between control cells and cells treated with 200 μM CW069 (MD 55 ± 7 and 53 ± 6 min, respectively, Figures 6A, 6B, and S7). Therefore, HSET inhibition by CW069 does not cause mitotic delay associated with inhibitors of KSP or CENP-E (Mayer et al., 1999), further indicating compound selectivity for HSET. As expected, cells treated with monastrol, a KSP-selective inhibitor, showed significant mitotic delay compared with control cells (MD 179 ± 68 and 53 ± 6 min, respectively; p < 0.001; Figures 6A, 6B, and S7). This effect was reversed to a large extent by cotreatment of CW069 with monastrol. The mean MD of cells transiting mitosis (60 ± 7 min) was significantly reduced compared with cells treated with monastrol alone (p < 0.001; Figures 6A, 6B, and S7), to a value comparable to that found for control cells. Importantly, these data show that inhibition of HSET using CW069 suppresses the mitotic delay observed in HeLa cells by inhibition of KSP with monastrol. It is noteworthy, however, that most (93%) of the HeLa cells treated with monastrol alone did not exit mitosis, but were arrested over the entire imaging period (Table 1). This is consistent with observations previously reported for KSP-depleted cells (Mountain et al., 1999). Cotreatment of CW069 with monastrol increased the percentage of cells exiting mitosis to 37% (Table 1), and hence suppressed mitotic arrest induced by KSP inhibition alone. These results further support the specificity of CW069 for HSET.

Immediately after time-lapse imaging, cells were fixed and antibodies against α-tubulin, CDK5RAP2, and DAPI were employed to visualize MTs, centrosomes, and DNA, respectively. This was undertaken to assess whether the monopolar mitotic spindles induced by KSP inhibition were also suppressed by cotreatment with the HSET inhibitor CW069. Control HeLa cells formed mainly bipolar spindles (92%), with two centrosomes positioned at the spindle poles on either side of the metaphase plate (Figure 6C; Table 1). Similar numbers of bipolar spindles were formed in CW069-treated cells (95%; Figure 6C; Table 1). Monastrol-treated cells almost exclusively formed monopolar spindles (97%) with one pole surrounded by DNA (Luo et al., 2004; Figure 6C; Table 1). Normal bipolar spindle formation was indeed restored for many cells (45%) when the HSET inhibitor CW069 was administered along with the KSP inhibitor monastrol (Figure 6C; Table 1).

### Differential Scanning Fluorimetry Experiments Indicate Specific Binding of Inhibitor CW069 to HSET

**Figure 7:**
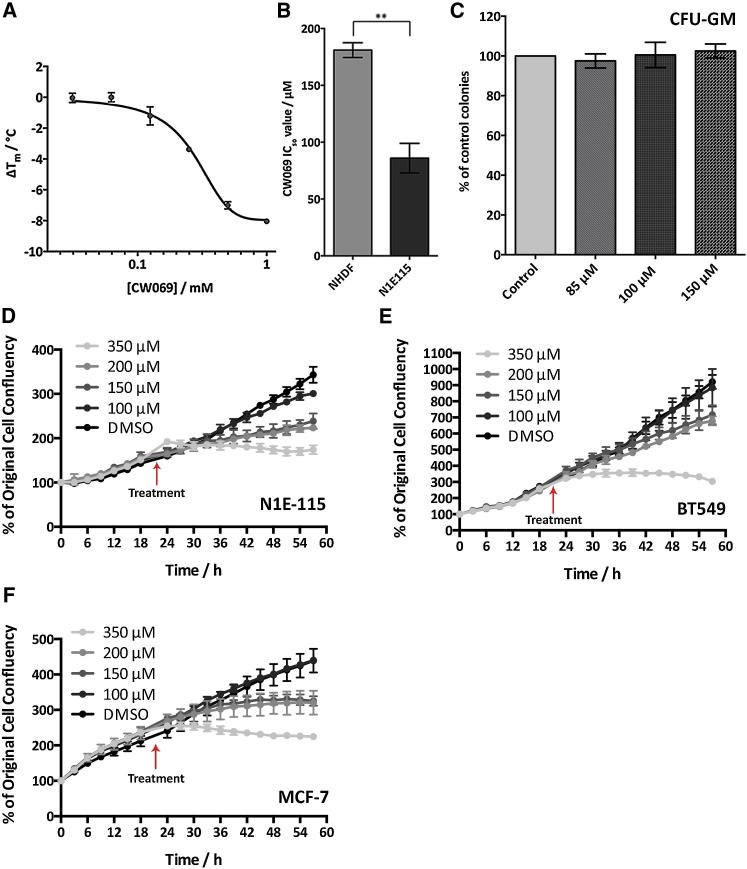
CW069 Binds in a Site-Specific Manner to HSET and Reduces Proliferation of Cancer Cells (A) DSF data for CW069 binding to full-length, N-terminal, 6His-tagged human HSET. CW069 concentrations: 0.03125 mM, 0.0625 mM, 0.125 mM, 0.25 mM, 0.5 mM, and 1 mM in 4% DMSO (n = 3). Data are represented as mean ± SD. CW069 binds in a dose-dependent manner to HSET and destabilizes it without causing the protein to denature. This indicates that binding is specific and electrostatic, not simply hydrophobic, in origin. (B) IC_50_ values derived from SRB growth-inhibition assays for CW069 in NHDF and N1E-115 cells. Concentrations from 0.3 μM to 400 μM (n = 3). Data are represented as mean ± SD. CW069 is significantly more cytotoxic in the N1E-115 cancer cells than in the NHDF cells. (C) Colony-formation assay using primary adult human bone marrow cells. Cells were treated with control (0.2% DMSO) or 85 μM, 100 μM, and 150 μM CW069 for 7 days (n = 3). Data are represented as mean ± SD. (D–F) IncyCyte live-cell imaging data over 58 hr for N1E-115 (D), BT549 (E), and MCF-7 (F) cancer cells. Cells were imaged for 21 hr before being treated with DMSO control (0.2%) or 100 μM, 150 μM, 200 μM, and 350 μM CW069 (n = 3). Data are represented as mean ± SD.

Specific binding of CW069 to HSET was also demonstrated via differential scanning fluorimetry (DSF), a technique that indicates the degree of protein stabilization/destabilization induced with ligand binding, as reflected by the ΔT_m_ value obtained (Niesen et al., 2007). DSF is an inexpensive and rapid method for identifying ligands of purified proteins, and relies on the difference in protein unfolding temperature in the presence and absence of a small molecule (Niesen et al., 2007). The unfolding temperature is measured by the increase in fluorescence of a dye with particular affinity for the hydrophobic regions of proteins, which become exposed upon unfolding (Niesen et al., 2007). The difference in the transition midpoint, ΔT_m_, in the presence of a ligand is related to its binding affinity; however, this is not a direct measure of the binding coefficient and therefore was not used to calculate the affinity of CW069 for HSET (Holdgate and Ward, 2005). CW069 had a maximum HSET ΔT_m_ of −8.0°C (Figure 7A). At lower inhibitor concentrations, the value decreased in magnitude in a dose-dependent manner and remained below zero, indicating that the compound destabilized HSET upon binding, without denaturing the protein (Figure 7A). These results demonstrate that CW069 binds to HSET in a site-specific manner, rather than simply through nonspecific hydrophobic interactions, confirming the in silico predictions described above.

### CW069 Inhibits Growth in Cancer Cells, but not in NHDF or Primary Human Bone Marrow Cells

We next assessed whether CW069 could selectively target proliferation in cancer cells. We used a combination of cell-growth-inhibition assays based on the Sulforhodamine-B (SRB) technique (Vichai and Kirtikara, 2006) and IncuCyte live-cell imaging. For this purpose, we used the mouse neuroblastoma cell line N1E-115, which has been reported to have a high frequency of centrosome amplification, and in which siRNA depletion of HSET has been shown to induce cell death by multipolar anaphases (Kwon et al., 2008). In addition, we tested NHDF cells with no centrosome amplification, where HSET inhibition was expected to have a significantly lower effect on viability. In agreement with our hypothesis (Figure 7B), CW069 was indeed significantly more potent against the N1E-115 cells than against the NHDF cells (IC_50_ 86 ± 10 μM and IC_50_ 181 ± 7 μM, respectively; p = 0.0016; Figure 7B). Additionally, the inhibitor did not reduce the clonogenic capacity of primary human bone marrow cells (Figure 7C). This assay is often used to assess possible neutropenia associated with mitotic inhibitors, and our data suggest that CW069 does not reduce proliferation of bone marrow cells and is unlikely to cause neutropenia. This further highlights that HSET inhibition, unlike that of KSP or CENP-E, provides a unique opportunity to selectively damage malignant cells without harming normal cells.

In order to ascertain whether CW069 could more broadly reduce proliferation in a range of cancer cells, we carried out IncuCyte live-cell imaging on N1E-115 cells and BT549 (with lower but marked centrosome amplification) and MCF-7 (with normal centrosome numbers) breast cancer cells. Cells were imaged for 21 hr prior to treatment with DMSO control (0.2%) and various concentrations of CW069, and then imaged for a further 36 hr. A reduction in cell proliferation induced by CW069 was indeed observed in the N1E-115 cells with the highest levels of centrosome amplification (p = 0.0192, comparing control treatment with 150 μM CW069; Figure 7D). However, growth inhibition was noted in all of the cancer cell lines (Figure 7). The growth-inhibitory effect of compound CW069 may not be solely due to its induction of aneuploidy through multipolar mitoses, as the compound does not alter normal bipolar division in MCF-7 cells but does reduce their proliferation. It could also be due to another direct or indirect effect, and the role of HSET in the survival of cancer cells with normal centrosome numbers remains to be fully understood. Taken together, therefore, these data suggest that inhibition of HSET using CW069 may be broadly applicable to a range of cancer cells with varying levels of centrosome amplification and is not restricted to the N1E-115 cancer cell line, although the strongest effect is observed in these cells, as predicted.

## Discussion

Almost a century ago, Theodor Boveri proposed that tumor cells differ from normal cells in that the former have a high incidence of centrosome amplification (Boveri, 2008). However, only recently have new therapeutic strategies been explored in an attempt to exploit this difference and the role of kinesins in mitosis. Intense interest in the field has led to the development of KSP and CENP-E inhibitors that have been tested clinically as treatments for human cancer (Huszar et al., 2009). Success has been limited because both motor proteins are essential for normal mitosis, and inhibition leads to mitotic arrest or delay and associated neutropenia toxicity in normal cells (Huszar et al., 2009). In contrast HSET is essential for the survival of cancer cells with centrosome amplification and has been shown to be dispensable in normal cells (Kwon et al., 2008). Hence, HSET inhibition offers a unique opportunity to selectively damage malignant cells with supernumerary centrosomes without affecting normal cells. In keeping with these findings, we report the discovery of an allosteric inhibitor of HSET, CW069, which does not disrupt mitosis in normal human fibroblast cells. In fact, CW069 induces multipolar mitosis exclusively in cancer cells with extra centrosomes, causing apoptosis via catastrophic aneuploidy. The increased multipolar mitoses induced in N1E-115 cells by the inhibitor CW069 recapitulates the phenotype described here and elsewhere (Kwon et al., 2008) for siRNA depletion of HSET.

The inhibitor also reduces centrosome clustering in cancer cells with a lower incidence of centrosome amplification, including BT549 and MDA-MB-231 breast cancer cells. This is consistent with recent reports that depletion of HSET in DNA-damage-repair-deficient cancer cells with low-level centrosome amplification may cause defects in the mitotic spindle (Kleylein-Sohn et al., 2012). CW069 did not alter bipolar mitosis in MCF-7 cancer cells; however, it did have an antiproliferative effect in these cells, as well as in BT549 and N1E-115 cancer cells.

Taken together, these data indicate the potential use of the HSET inhibitor CW069 across a range of human cancers. Moreover, CW069, unlike KSP or CENP-E inhibitors, does not cause mitotic delay in HeLa cells with normal centrosome numbers, nor does it decrease the clonogenic capacity of primary adult human bone marrow cells. Thus, CW069 may not cause neutropenia toxicity in normal cells. It is anticipated that HSET inhibition could have a greater therapeutic margin than KSP or CENP-E inhibition, and we have described an allosteric inhibitor of HSET that reduces centrosome clustering but does not induce the mitotic phenotypes associated with inhibition of KSP or CENP-E. This selectivity for HSET is consistent with our computational model, which indicates that the HSET loop 5 displays a dynamic conformational selection for CW069 that cannot be achieved by the closely related KSP. In fact, the motor antagonism between plus-end-directed KSP and minus-end-directed HSET is understood to be responsible for establishing a proper bipolar spindle during mitosis (Mountain et al., 1999), and KSP inhibition has been shown to result in monopolar spindle formation as well as mitotic delay (Gatlin and Bloom, 2010; Mayer et al., 1999).

## Significance

**Over the past decade, new cancer therapeutic strategies have been designed to exploit the role of kinesin motor proteins in MT dynamics and mitosis (****Huszar et al., 2009****). Inhibitors of the MT motor proteins KSP and CENP-E, which are both essential for mitosis in normal cells, have been limited by toxic side effects in normal cells (****Huszar et al., 2009****). However, inhibition of HSET exploits the higher incidence of centrosome amplification in cancer cells compared with normal cells and thus provides a unique opportunity to selectively damage malignant cells with supernumerary centrosomes (****Kwon et al., 2008****). With this in mind, we designed an allosteric inhibitor of HSET, CW069, which does not disrupt division in normal cells or reduce clonogenic capacity in primary adult human bone marrow cells, but leads to multipolar mitosis in cancer cells with extra centrosomes, causing apoptosis via catastrophic aneuploidy. CW069 also reduces cell growth and centrosome clustering in cancer cells with a lower incidence of centrosome amplification, including BT549 and MDA-MB-231 breast cancer cells, indicating that the inhibitor could be broadly applicable to a range of human cancers. Additionally, our data indicate that CW069 does not cause neutropenia in normal cells, a side effect that is commonly observed with inhibition of KSP and CENP-E. In accordance with the opposing activities of plus-end-directed KSP and minus-end-directed HSET, CW069 treatment suppresses the monopolar phenotype and mitotic arrest induced by inhibition of KSP in HeLa cells. In summary, CW069 not only represents an advance toward new cancer therapeutics, but also offers researchers a unique tool to unveil the full details of HSET function in mitosis.**

## Experimental Procedures

### Chemogenomics Compound Selection

Given the high degree of sequence similarity between the motor domains of HSET and KSP, for computational compound selection we employed the chemogenomics principle that similar proteins bind similar ligands (Bender et al., 2007). We selected 466 compounds that are active against KSP from ChEMBL (Gaulton et al., 2012) and created a Support Vector Machine model for bioactivity based on ECFP_6 fingerprints in PipelinePilot Student Edition 6.1. In addition, we employed a deselection model that ruled out compounds that could potentially display off-target effects. This model was employed to screen the ZINC database for potential HSET inhibitors, and the top 200 compounds were clustered. Manual selection (biasing compound diversity as well as desirable chemical functional groups) led to a selection of the 50 highest-scoring compounds for enzymatic in vitro screening against HSET.

### In Vitro Enzymatic ATPase Assay

The protocol was optimized for use with full-length, N-terminal, 6His-tagged HSET and KSP, and measured the MT-stimulated activity of the proteins. Inhibition of the Gsp synthetase activity of HSET/KSP was observed spectrophotometrically by coupling the hydrolysis of ATP to oxidation of NADH via pyruvate kinase/lactate dehydrogenase reactions. The assay was initiated by adding purified Gsp synthetase/amidase (12.8 nM) to an assay mixture containing the following components (final concentration): 6 nM protein, 0.07 mg/ml MTs (University Biologicals), 1.56 mM glutathione, 10 mM spermidine, 2 mM ATP, 2.7 mM MgCI, 1 mM phospho(enol)-pyruvate, 0.2 mM NADH, 50 μg/ml lactate dehydrogenase, 100 μg/ml pyruvate kinase, and various concentrations of inhibitor all in 50 mM Na PIPES (pH 6.8) at 37°C. The ADP-Glo detection assay (Promega) was performed as described in the manufacturer’s instructions. All compound additions were performed using a multidrop BioMek Nxp (Beckman Coulter). Plates were read using a Pherastar microplate reader (BMG Labtech).

### Growth Inhibition

NHDF cells were obtained from PromoCell, and the N1E-115 and HeLa cells were obtained from ATCC/LGC Standards. The BT549, MCF-7 and MDA-MB-231 cells were kind gifts from the Caldas laboratory (CRUK Cambridge Institute) and were originally obtained from ATCC/LGC. All human cells were verified by STR genotyping and all tested negative for mycoplasma. Cells were cultured in Dulbecco’s modified Eagle’s medium (DMEM) supplemented with 10% fetal calf serum (FCS) at 37°C and 5% CO_2_. All compounds used in the Sulforhodamine B colorimetric (SRB) assay (Vichai and Kirtikara, 2006) were dissolved in DMSO and diluted in culture medium to a final concentration of 0.2% DMSO. For the SRB assay and live-cell imaging (using the IncuCyte Kinetic Live Cell Imaging System; Select Science), cells were seeded in 96-well plates at a density of 2,500 cells per well. After 24 hr, the cells were treated with compound for 72 hr, with triplicate wells for each concentration. For the SRB assay, the cells were then fixed with trichloroacetic acid (TCA) and stained with SRB. Fluorescence was quantified using an Infinite 200 PRO plate-reader (Tecan) at a wavelength of 545 nm. Compound-treated wells were compared with solvent control wells and the concentration of compound that resulted in 50% of the solvent-control cell growth was designated as the IC_50_ concentration, calculated using Graphpad PRISM 6. At least three biological replicates were performed for each assay.

For live-cell imaging, plated cells were imaged prior to treatment for 24 hr and then after treatment for 72 hr. Images were gathered every 3 hr. Data were analyzed as the percentage of original cell confluency in order to directly compare each compound treatment with the DMSO control.

### Immunofluorescence

Cells were plated at a seeding density of 1.2 × 10^4^ cells per well in Ibidi eight-well chamber slides, incubated for 24 hr, and then treated with compound for 3 hr. Prewarmed 4% paraformaldehyde in PBS was then added to each well to fix the cells before washing with PBS. Permeabilization and blocking was performed by incubating the slides with 1% BSA, 0.3% Triton X-100, 5% goat serum in PBS (PBS/B/0.3%T+5%GS) for 1 hr while shaking them at room temperature (RT). The slides were incubated with primary antibody solutions (mouse monoclonal anti-α-Tubulin [catalog number T9026; Sigma-Aldrich] and rabbit anti-CDK5RAP2 [catalog number A300-554A; Bethyl Laboratories]) at 4°C for 16 hr. The slides were washed four times with PBS/B/0.3%T+5%GS and then incubated with secondary antibody solutions (anti-rabbit IgG Alexa Fluor 488 [catalog number A21206] and anti-mouse IgG Alexa Fluor 647 [catalog number A20990]; Invitrogen) and DAPI (catalog number D3571; Invitrogen) in deionized water (300 nM) in the dark at RT for 1 hr. After four washes with PBS/B/0.3%T+5%GS, the solution was replaced with PBS and the slides were stored at 4°C until imaging was performed. Images were gathered with the use of an ICys Research Imaging Cytometer (CompuCyte) or a tandem confocal microscope (Leica).

### Time-Lapse Microscopy

Cells were synchronized in G2-M by incubation at 37°C and 5% CO_2_ for 24 hr with 9 μM of the CDK1 inhibitor RO-3306 (Merck). Cells were released by removal of the RO-3306 and four washings of 10 min each, at 37°C, with prewarmed culture media + 10% FBS. N1E-115 cells were transfected with siRNA (Supplemental Experimental Procedures) and imaged from 6 hr after transfection. Some RO-3306-synchronized cells were released with 200 μM CW069, 100 μM monastrol, or 0.2% DMSO, and imaging started immediately or 1 hr after treatment. Imaging was carried out for 24 hr, or for 4 hr in the case of the CW069/monastrol cotreatment. Imaging was conducted every 5 min in a humidified chamber with 37°C and 5% CO_2_, using a Nikon Eclipse TE2000-E microscope, and analyzed with NIS-Elements software (Nikon, Tokyo, Japan). The morphology of cells in mitosis and the mitotic fate (bipolar division, multipolar division, or cell death) were analyzed.

### Statistical Analyses

The errors reported were calculated as SDs, and p values were calculated using two-way ANOVA when appropriate or multiple two-tailed, unpaired t tests in Graphpad Prism 6 software, correcting for multiple comparisons using Holm-Sidak compensation. For statistical significance, ^∗^p ≤ 0.05, ^∗∗^p ≤ 0.01, and ^∗∗∗^p ≤ 0.001.

## Author Contributions

C.A.W. synthesized and characterized all of the compounds, including CW069. C.A.W. designed, conducted, and analyzed all of the biological experiments except for the DSF assay, which was carried out by P.O., and the enzymatic ADP-Glo assay, which was designed by M.R. A.B., P.J.B., and O. Korb conducted the molecular modeling with input from C.A.W. The manuscript was written by C.A.W. F.M.R., R.M.M., J.R., O. Kern, F.G., D.I.J., and S.V.L. added to the scientific content of the paper through helpful discussions and review of the final manuscript.

## Figures and Tables

**Table 1:** Simultaneous HSET and KSP Inhibition Increases Centrosome Separation and Suppresses Mitotic Arrest Induced by Inhibition of KSP Only

Treatment	Mitotic Arrest (%)^a^	Mitotic Exit (%)^a^	Monopolar Spindle (%)^b^	Bipolar Spindle (%)^b^
DMSO	3	97	8	92
CW069	4	96	5	95
Monastrol	93	7	97	3
CW069+monastrol	63	37	55	45

Related to Figure 6.

a Percentage of >200 cells (for each treatment) that entered mitosis >60 min after treatment.

b Percentage of 40 mitotic cells, chosen at random, for each treatment.

## Supplemental Information

Document S1. Supplemental Experimental Procedures and Figures S1–S5

Movie S1. Representative Time-Lapse Video of an MCF-7 Cell Treated with DMSO Control Undergoing Bipolar Division

Movie S2. Representative Time-Lapse Video of an MCF-7 Cell Treated with 200 μM CW069 Undergoing Bipolar Division

Movie S3. Time-Lapse Video Showing HeLa Cells Progressing through Mitosis after 60 Min Treatment with DMSO Cells were imaged for 360 min.

Movie S4. Time-Lapse Video Showing HeLa Cells Progressing through Mitosis after 60 Min Treatment with 200 μM CW069 Cells were imaged for 360 min.

Movie S5. Time-Lapse Video Showing HeLa Cells Progressing through Mitosis after 60 Min Treatment with 100 μM Monastrol Cells were imaged for 360 min.

Movie S6. Time-Lapse Video Showing HeLa Cells Progressing through Mitosis after 60 Min Treatment with 200 μM CW069 + 100 μM Monastrol Cells were imaged for 360 min.
