# Supplementary material for: Design, Synthesis, and Biological Evaluation of an Allosteric Inhibitor of HSET that Targets Cancer Cells with Supernumerary Centrosomes
Source: Chem Biol. 2013 Nov 21;20(11):1399–410. doi: 10.1016/j.chembiol.2013.09.012 (PMC3898838; doi:10.1016/j.chembiol.2013.09.012)
Supplement: Document S1. Supplemental Experimental Procedures and Figures S1–S5 [file mmc1.pdf]

Chemistry & Biology, Volume *20*

Supplemental Information

Design, Synthesis, and Biological Evaluation of an Allosteric Inhibitor of HSET that Targets Cancer Cells with Supernumerary Centrosomes

Ciorsdaidh A. Watts, Frances M. Richards, Andreas Bender, Peter J. Bond, Oliver Korb, Oliver Kern, Michelle Riddick, Paul Owen, Rebecca M. Myers, Jordan Raff, Fanni Gergely, Duncan I. Jodrell, and Steven V. Ley

SUPPLEMENTARY DATA

S1A. Chemical structures of synthetic analogs of compound 2 tested *in vitro* for activity against HSET and KSP proteins. **S1B.** Chemical structures of commercial analogs of tested for activity against HSET and KSP proteins. **S1C.** Original synthetic route used to synthesize HSET inhibitor CW069. *Reagents and conditions*: (a) T3P coupling agent, DIPEA, CH_2Cl_2, rt, 16 h, 26%; (b) TFA, CH_2Cl_2, rt, 1 h, 78%; (c) (i) NEt_3, THF, (ii) benzaldehyde, THF, (iii) $NaCNBH_3$, THF, rt, 16 h, 81%. **Figure S1** is associated with main text **Figure 1**.

A

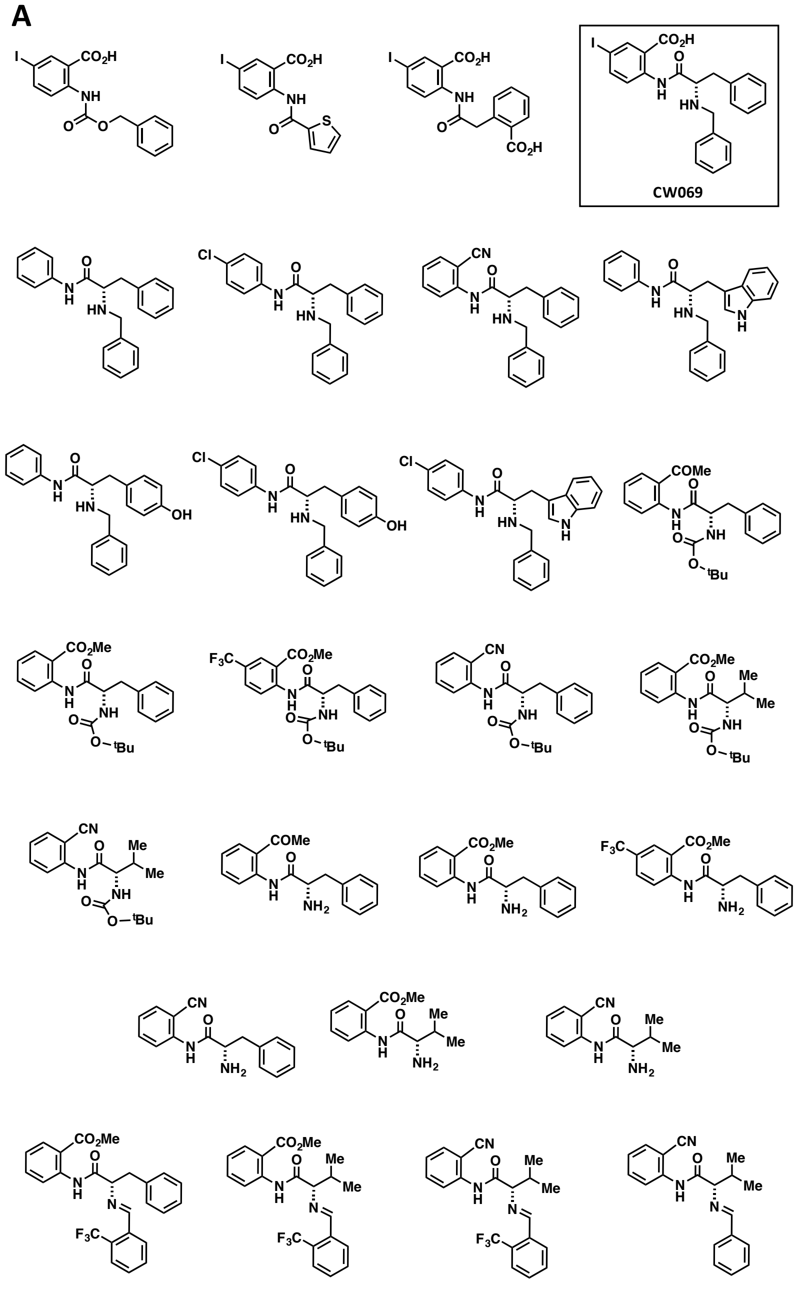

B

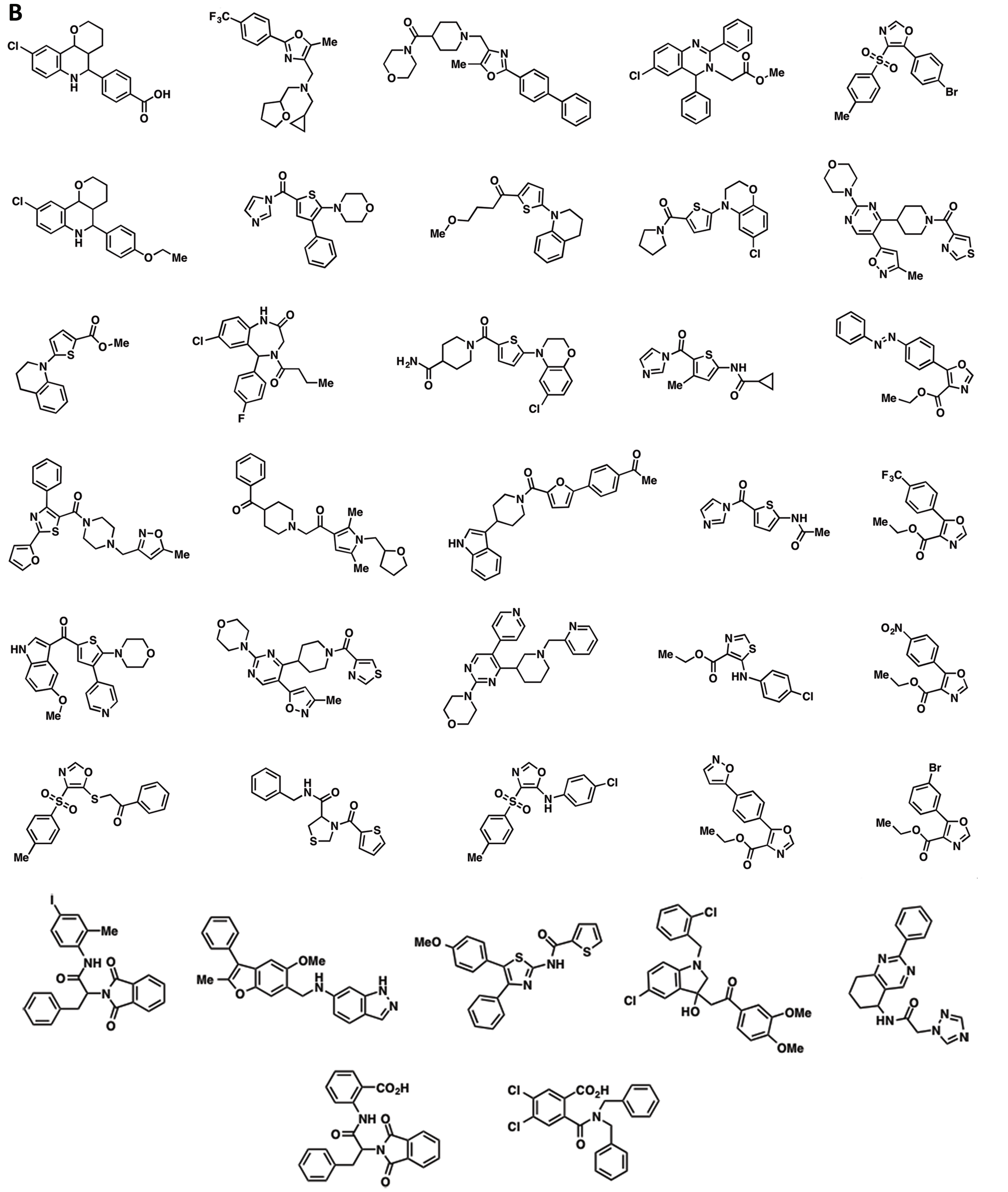

C

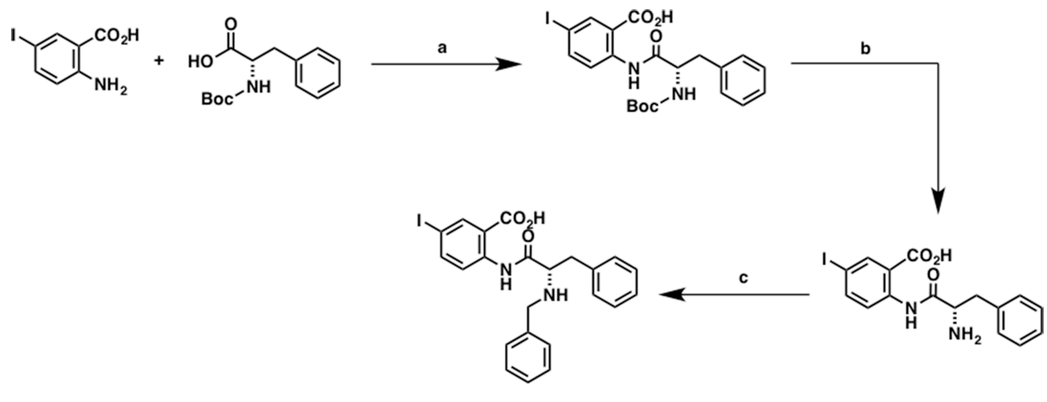

S2. Dose response curve for CW069 against full-length HSET protein in the ADP-Glo assay, n=4 (top). Dose response curve for CW069 against full-length KSP protein in the ADP-Glo assay, n=4, representative graph (bottom). **Figure S2** is associated with main text **Figure 2**.

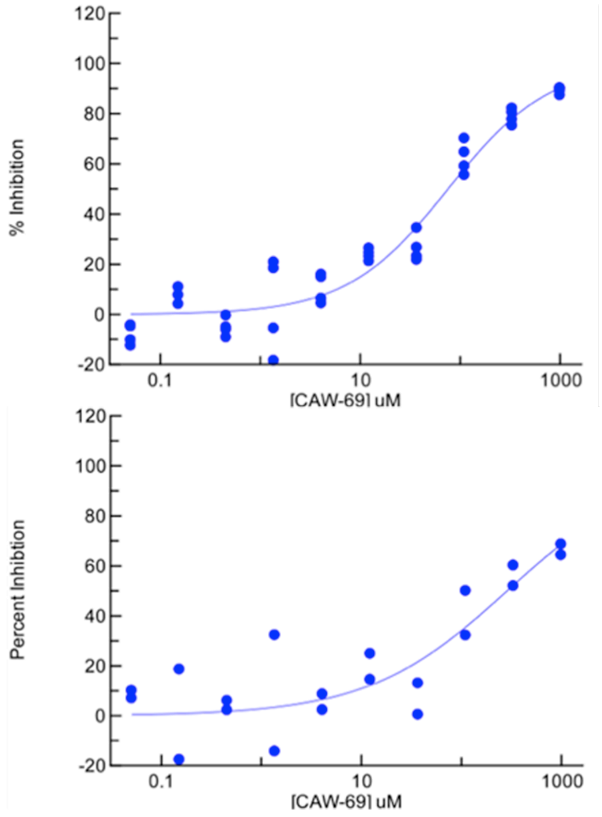

S3. *In silico* model showing the predicted weak interactions between the enantiomer of CW069 (right panel) with the HSET loop 5 cleft compared to the optimal interactions between CW069 itself and HSET (left panel). **Figure S3** is associated with main text **Figure 3**.

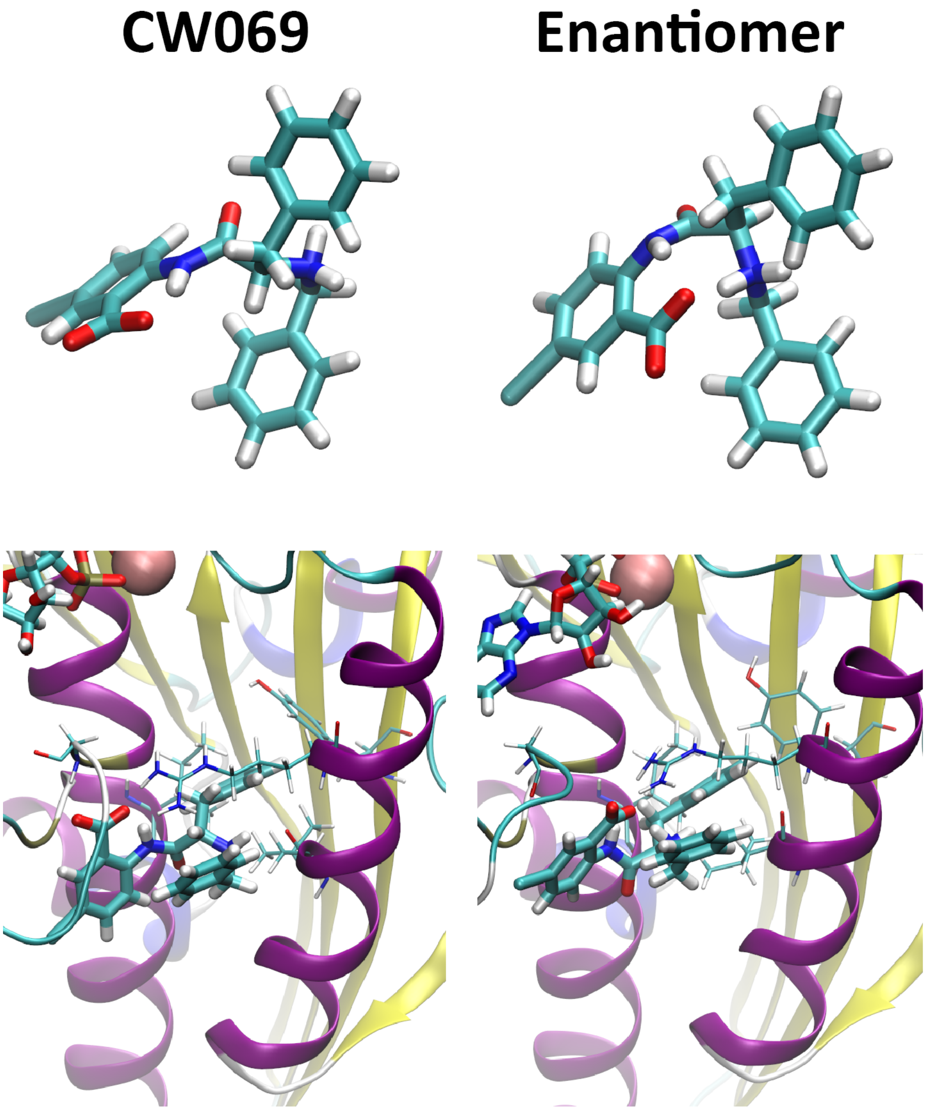

S4A. Immunofluorescence data for MDA-MB-231 cells. **S4B.** Immunofluorescence data for BT549 cells. **S4C.** Immunofluorescence data for MCF-7 cells. Cells were treated with DMSO control (0.2%), 100 μM and 200 μM CW069 for 2.5 h, MC = mitotic cells, CE = centrosomes, Mono = monopolar, Bi = bipolar, Multi = multipolar, n=3 wells from each of which at least 200 mitotic cells were scored, data represented as mean ± SD. **Figure S4** is associated with main text **Figure 4**.

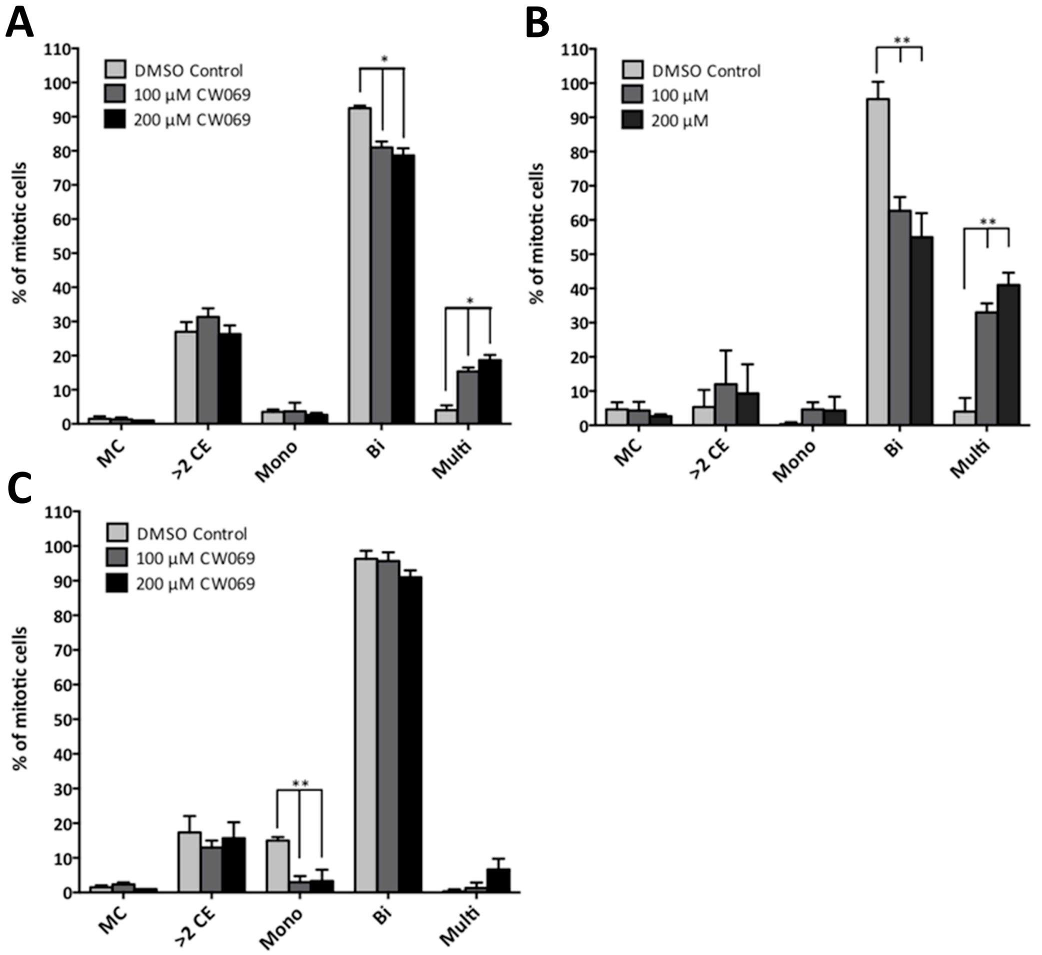

S5A. Western blot showing sustained HSET knockdown over 48 h after siRNA transfection of N1E-115 mouse neuroblastoma cells. **S5B.** Western blot showing sustained HSET knockdown over 48 h after siRNA transfection of BT549 human breast cancer cells. **Figure S5** is associated with main text **Figure 5**.

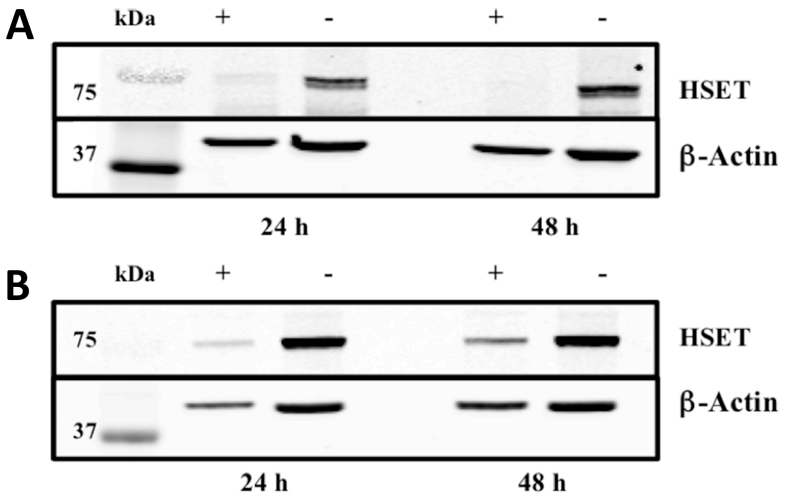

S6. Representative time-lapse video of an MCF-7 cell treated with (A) DMSO control (0.2%) undergoing bipolar division, and (B) 200 μM CW069. Cells undergo bipolar division (see accompanying video files). **Figure S6** is associated with main text **Figure 5**.

S7. Time-lapse videos showing HeLa cells progressing through mitosis after 60 minutes treatment with (A) DMSO (0.2%), (B) 200 μM CW069, (C) 100 μM monastrol, and (D) 200 μM CW069 + 100 μM monastrol. Cells were imaged for 360 minutes (see accompanying video files). **Figure S7** is associated with main text **Figure 6**.

SUPPLEMENTARY EXPERIMENTAL PROCEDURES

Computational Model Building. In order to generate potential binding mode hypotheses of CW069 a two-step ligand-based alignment approach has been used. Compound 3 was first aligned onto a set of known KSP inhibitors and consecutively CW069 onto the resulting superimposed conformation of 3.

The initial conformations of CW069 and 3 were created using CORINA (Sadowski and Gasteiger, 1993). Based on fingerprint similarity, 7 KSP compounds (ligands binding to the allosteric pockets of PDB 2FL2, 2FL6, 2PG2, 2Q2Y, 2Q2Z, 2UYM and 3CJO) were selected and their corresponding binding sites were superimposed using Relibase (Hendlich et al., 2003). The resulting superimposition of the seven ligands was used as the target to overlay compound 3 onto. As compound 3 contains several rotatable bonds, a flexible ligand superimposition method was used to generate alignment hypotheses (Korb et al., 2010). The HSET protein structure (PDB 2REP) was aligned onto the KSP protein structures to obtain ligand-based predicted binding modes of 3 and CW069 in HSET. The predicted complex conformations were then subjected to energy minimization.

Full-length models of the motor domains of Mg^{2+}.ADP-bound HSET (PDB 2REP) and KSP (PDB 1II6), and of KSP in complex with monastrol and Mg^{2+}.ADP (PDB 1Q0B), were built on the basis of X-ray structures following reconstruction of several disordered loops that were missing in the crystallographic density. For HSET, these were residues 307-309, 337-342, 352-368, 477-484, 491-493, 529-533, 571-594, and 662-663. For monastrol-bound and free KSP, these were residues 272-287 and 274-280, respectively. Loops were reconstructed and subsequently refined using the DOPE module in the Modeller suite (Eswar et al., 2006).

Optimization of Protein-CW069 Complexes. Optimization of each Mg^{2+}.ADP-complexed protein in the presence of compound CW069 was carried out using a multi-step protocol. Initially, the conformation of CW069 was constrained, and several thousand steps of adopted basis Newton-Raphson (ABNR) minimization were performed until protein-ligand interaction energies reached convergence. Subsequently, the constraints on the ligand were released, and several further thousand ABNR steps were carried out. Following convergence, and a final, short steepest descent minimization step, the protein-ligand interaction energy and its component contributions were recorded. Each protein-ligand complex was optimized in the CHARMM program (Brooks et al., 2009), using the CHARMM27/CMAP protein parameters, and the CHARMM CGenFF general force field for compound CW069 (Vanommeslaeghe et al., 2010). Non-bonded interactions were treated with an atom-based cutoff of 1.4 nm. Electrostatics used a distance-dependent dielectric, with a shifted potential going to zero beyond 1.2 nm. Van-der-Waals Forces were switched off from 1.0 nm to 1.2 nm.

Molecular Dynamics Simulations of Inhibitor-Free Motor Domains. The ionizable groups of each protein model were assigned their most probable charged states at neutral pH. Each protein in complex with Mg^{2+}.ADP was solvated with TIP3P water and a ~0.1 M concentration of NaCl in a truncated octahedral periodic box of dimension ~9-10 nm. Overlapping solvent molecules were removed, resulting in systems containing ~60,000-80,000 atoms. At each stage of system setup, steepest descent energy minimization was performed to relax the protein geometry and to remove steric clashes between protein, cofactor, solvent and ions. Each system was

equilibrated over 2 ns, during which position restraints, applied to all non-hydrogen protein atoms except modeled loop regions, were gradually removed to relax the protein structure and solvent.

A total of 1 μs of production simulation sampling was generated for the three protein systems, each split into ten sets of independent 100 ns trajectories to ensure efficient exploration of conformational space.

All simulations were performed using GROMACS (Hess et al., 2008) version 4.5 (Bjelkmar et al., 2010). The protein was treated using the CHARMM22/CMAP force field (MacKerell et al., 1998). Equations of motion were integrated using the leapfrog method with a 2 fs time step, and the LINCS algorithm was used to constrain bond lengths (Hess et al., 1997). Electrostatic interactions were computed using the Particle-Mesh-Ewald (PME) algorithm (Essmann et al., 1995) and the real-space sum was cut off at 12 Å. Van-der-Waals Interactions were switched off between 10 Å and 12 Å and the neighbor list was updated every 10 steps. Simulations were performed using conditions of constant temperature (300 K) and pressure (1 atm) *via* the Bussi thermostat (Bussi et al., 2007), and isotropic pressure-coupling using the Parrinello-Rahman barostat (Parrinello and Rahman, 1981) with a coupling constant of 1 ps. Clustering analysis was performed using GROMACS. Visual analysis was performed using VMD (Humphrey et al., 1996).

Synthetic Chemistry. All non-aqueous reactions were conducted in oven-dried (200 °C) glassware under an inert atmosphere of dry argon. Solvents were purified and dried using standard methods prior to use: THF and Et_2O by distillation from calcium hydride and lithium aluminium hydride; CH_2Cl_2, toluene and acetonitrile by

distillation from calcium hydride. Flash column chromatography was performed under pressure with silica gel 60 (particle size 40-63 nm) using gradient elutions. All solvents used for chromatographic purification were distilled prior to use. Analytical TLC was performed using silica gel 60 F_{254} pre-coated glass backed plates and visualised by ultraviolet radiation (254 nm), and/or by potassium permanganate stain. 'Hexanes' refers to the use of b.p. 40-60 °C petroleum ether eluent.

^{1}H NMR spectra were recorded on a Bruker DPX-400 (400 MHz) spectrometer. Chemical shifts are reported in ppm with the resonance resulting from incomplete deuteration of the solvent as the internal standard ($CDCl_3$: 7.26 ppm or d_6-DMSO: 2.50 ppm). Data are reported as follows: chemical shift δ/ppm (multiplicity (s = singlet, d = doublet, t = triplet, q = quartet, br = broad, m = multiplet), coupling constants J/Hz, assignment, and integration). ^{13}C NMR spectra were recorded on a Bruker DPX-400 (100 MHz) spectrometer with complete proton decoupling. Chemical shifts are reported in ppm with the solvent resonance as the internal standard ($^{13}CDCl_3$: 77.0 ppm, t or ^{13}C-d_6-DMSO: 39.5 ppm, septet).

High-resolution mass spectrometry (HRMS) was performed on a Waters Micromass LCT spectrometer using electrospray ionisation and Micromass MS software. Infrared spectra were recorded as thin films on a Perkin-Elmer Spectrum One FT-IR spectrometer and only selected peaks are reported. Optical rotations were measured using a Perkin-Elmer polarimeter (Na/Halogen 589 nm). Elemental analyses were performed by the microanalysis service in the Department of Chemistry

Optimization of the Synthesis of CW069. The original synthetic route toward CW069 was a three-step process in which the key peptide-bond formation (Figure S1C) was achieved using the coupling agent propylphosphonic anhydride solution (T3P). However, poor yields were routinely observed for this reaction (26%), possibly due to an undesired side-reaction of the carboxylic acid moiety. The deprotection and reductive amination steps progressed well however (78% and 81%, respectively). The reductive amination proceeds through formation of an imine species followed by subsequent reduction by $NaCNBH_3$, as described by Grenga (Grenga et al., 2009). Because our original synthetic route proved inefficient, a variety of alternative reaction conditions were explored to afford the desired peptide product in good yield. A Buchwald-Hartwig C-N bond-formation was eventually chosen as the most effective reaction in this case (main text Figure 1C). Good conversion to the peptide product (90%) was only achieved using these specific reaction conditions. A screen identified $Pd_2(dba)_3$/Xantphos as the best catalyst-ligand combination, with conversion increasing in parallel with catalytic loading.

(*S*)-2-(2-((*tert*-Butoxycarbonyl)amino)-3-phenylpropanamido)-5-iodobenzoic acid CW067

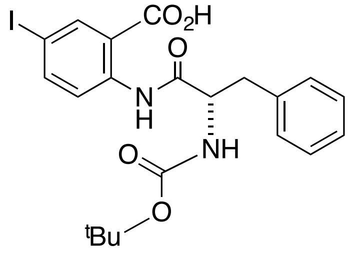

2-Amino-5-iodobenzoic acid (250 mg, 0.950 mmol) and N-Boc-L-Phenylalanine (252 mg, 0.950 mmol) were dissolved in CH_2Cl_2 (30 mL). DIPEA (340 μL, 1.98 mmol)

was added and the reaction was cooled to 0 °C. T3P (680 μL of a 50 wt% solution in EtOAc, 1.14 mmol) was added dropwise over 10 mins, the reaction was allowed to warm to rt, and stirred for 16 h. The mixture was partitioned between water (30 mL) and CH_2Cl_2 (30 mL). The aqueous phase was extracted with CH_2Cl_2 (3 × 30 mL) and the combined organics were washed with water (15 mL), dried over $MgSO_4$, filtered and concentrated *in vacuo*. Purification was achieved by flash silica chromatography (20% EtOAc in hexanes). Fractions were combined and the solvent was evaporated *in vacuo* to afford CW067 as a white solid (128 mg, 0.251 mmol, 26%).

R_f (20% EtOAc in hexanes) 0.44.

m.p. 128 °C.

$[\alpha]_D^{28.2}$ = –46 (1.0, $CDCl_3$).

EA, found: C, 49.42; H, 4.14; N, 5.07, $C_{21}H_{23}IN_2O_5$ requires C, 49.42; H, 4.54; N, 5.29.

IR υ_{max}(film)/cm^{-1} 3363, 1765, 1643, 1595, 1497, 1366, 1246, 1161, 836, 700.

^{1}H NMR (400 MHz; $CDCl_3$) δ ppm: 1.42 (s, CH_3, 9H), 3.17 (dd, *J* 12.9, 5.2, CH_{2a}, 1H), 3.30 (dd, *J* 12.8, 5.2, CH_{2b}, 1H), 4.92 (d, *J* 5.2, C*H*, 1H), 5.19 (d, *J* 5.2, NH, 1H), 7.12 (d, *J* 8.6, Ar*H*, 1H), 7.12 (d, *J* 6.3, Ar*H*, 2H), 7.23 (m, Ar*H*, 3H), 8.06 (d, *J* 8.4, Ar*H*, 1H), 8.51 (d, *J* 1.5, Ar*H*, 1H).

^{13}C NMR (100 MHz, $CDCl_3$) δ ppm: 38.9, 54.1, 61.3, 92.7, 118.3, 123.4, 125.6, 126.2, 127.1, 127.3, 128.6, 128.9, 129.3, 135.4, 136.2, 137.2, 145.0, 169.6, 172.4.

HRMS (ESI+) calculated for $C_{21}H_{24}IN_2O_5$ $[M+H]^+$ 511.0730, found 511.0746.

(*S*)-2-(2-Amino-3-phenylpropanamido)-5-iodobenzoic acid CW068

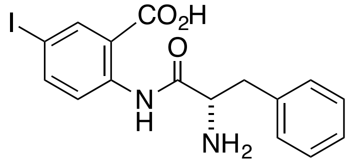

(*S*)-2-(2-((*tert*-Butoxycarbonyl)amino)-3-phenylpropanamido)5-iodobenzoic acid CW067 (120 mg, 0.235 mmol) was dissolved in CH_2Cl_2 (1.5 mL). TFA (1.5 mL) was then added dropwise and the reaction mixture was stirred at rt for 1 h. The mixture was evaporated to dryness *in vacuo*. Purification was by flash silica chromatography (80-100% EtOAc in hexane). Fractions were combined and the solvent was evaporated *in vacuo* to afford CW068 (96 mg, 0.183 mmol, 78%) as a white solid.

R_f (5% MeOH in CH_2Cl_2) 0.29.

m.p. 126 °C.

$[\alpha]_D^{28.1}$ = +31 (1.0, MeOH).

IR υ_{max}(film)/cm^{-1} 3695, 3425, 1659, 1509, 1382, 1292, 1187, 921, 865, 798.

^{1}H NMR (400 MHz; *d_6*-DMSO) δ ppm: 3.15 (m, C*H_2*, 2H), 4.35 (s, C*H*, 1H), 7.27 (m, Ar*H*, 4H), 7.94 (dd, *J* 8.8, 2.1 Ar*H*, 1H), 8.02 (d, *J* 8.8, Ar*H*, 1H), 8.16 (d, *J* 2.1, Ar*H*, 1H), 8.44 (s, N*H*, 1H), 11.09 (s, br, CO_2H, 1H).

^{13}C NMR (100 MHz, *d_6*-DMSO) δ ppm: 37.0, 55.2, 87.9, 120.8, 123.3, 127.4, 128.8, 129.4, 134.6, 138.3, 139.1, 142.2, 167.2, 167.5.

HRMS (ESI+) calculated for $C_{16}H_{16}IN_2O_3$ $[M+H]^+$ 411.0206, found 411.0207.

(*S*)-2-(2-(Benzylamino)-3-phenylpropanamido)-5-iodobenzoic acid CW069

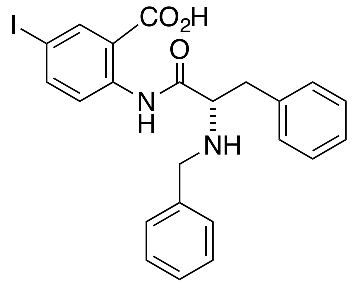

(*S*)-2-(2-Amino-3-phenylpropanamido)-5-iodo-benzoic acid CW068 (90 mg, 0.219 mmol) was dissolved in dry THF (2 mL). Triethylamine (30 μL, 0.215 mmol) was added and the reaction was stirred at rt for 15 mins. Benzaldehyde (23 μL, 0.226 mmol) was added dropwise and the reaction was stirred at rt for a further 2 h. The solution was cooled to 0 °C and $NaCNBH_3$ (28 mg, 0.446 mmol) was added dropwise and the reaction was stirred for a further 16 h at rt. The mixture was partitioned between water (10 mL) and CH_2Cl_2 (10 mL). The aqueous phase was extracted with CH_2Cl_2 (3 × 10 mL) and the combined organics were washed with brine (15 mL), dried over $MgSO_4$, filtered and concentrated *in vacuo*. Purification was achieved by flash silica chromatography (5-10% MeOH in CH_2Cl_2). Fractions were combined and the solvent was evaporated *in vacuo* to afford CW069 as a white solid (89 mg, 0.178 mmol, 81%).

R*f* (10% MeOH in CH_2Cl_2) 0.43.

m.p. 156 °C.

$[\alpha]_D^{27.8}$ = –57 (1.0, $CDCl_3$).

EA, found: C, 55.46; H, 4.21; N, 5.34, $C_{23}H_{21}IN_2O_3$ requires C, 55.41; H, 4.23; N, 5.60.

IR υ_{max}(film)/cm^{-1} 3087, 1608, 1574, 1364, 1291, 1087, 880, 824.

^{1}H NMR (400 MHz; *d$_6$*-DMSO) δ ppm: 2.89 (dd, *J* 13.5, 7.5, C*H*$_{2a}$, 1H), 3.02 (dd, *J*

13.5, 6.0, CH_{2b}, 1H) 3.37 (dd, *J* 7.5, 6.0, C*H*, 1H), 3.57 (d, *J* 14.0, CH_{2aa}, 1H), 3.76 (d, *J* 14.0, CH_{2bb}, 1H), 7.20 (m, Ar*H*, 10H), 7.72 (d, *J* 7.5, Ar*H*, 1H), 8.25 (s, Ar*H*, 1H), 8.36 (d, *J* 8.5, Ar*H*, 1H), 13.27 (s, br, CO_2H, 1H).

^{13}C NMR (100 MHz, d_6-DMSO) δ ppm: 51.5, 64.4, 85.6, 121.3, 121.5, 126.5, 126.9, 127.9, 128.2, 128.3, 128.4, 129.3, 137.8, 137.9, 139.5, 139.7, 140.1, 140.2, 167.9, 172.4.

HRMS (ESI+) calculated for $C_{23}H_{22}IN_2O_3$ $[M+H]^+$ 501.0675, found 501.0694.

Purification of HSET and KSP Proteins. Full length (FL) HSET, motor domain HSET (MD), and FL KSP constructs were created at Cancer Research Technology Ltd by Leon Pang. Briefly, HSET DNA encoding FL (M1-K673), or MD (L307-C663) was cloned into a pFastBac1 plasmid (engineered to contain an N-terminal 6His tag), under the polyhedrin promoter. This plasmid was used to produce bacmids for infection of Sf9 cells, inducing synthesis of 6His-tagged HSET FL and MD protein. Protein was expressed by infection of cells for 48 h at a viral titre of 5×10^8 and a multiplicity of infection (MOI) of 5, cells were then harvested by centrifugation and stored at -80 °C. Purification was carried out by Louise Tonkin at Cancer Research Technology Ltd. Pellets of Sf9 cells were thawed and re-suspended in 500 mL 20 mᴍ Tris pH 7.5, 0.5 ᴍ NaCl, 5% glycerol, 5 mᴍ imidazole, 1 mᴍ of the reducing agent tris(2-carboxyethyl)phosphine TCEP, 0.5% NP-40, and benzonase nuclease. The cells (growth volume 0.5 L) were lysed by mixing at 4 °C for 30 mins and centrifuged to separate soluble/insoluble material (16000 rpm, 1 h). The supernatant was then mixed with 10 mL NiNTA beads equilibrated with wash buffer (20 mᴍ Tris pH 7.5, 0.5 ᴍ NaCl, 5% glycerol, 15 mᴍ imidazole, 2 mᴍ TCEP)

and rolled at 4 °C for 2 h. The beads were harvested by centrifugation and washed with 2 × 100 mL 2 mᴍ Tris pH 7.5, 0.5 ᴍ NaCl, 5% glycerol and 2 mᴍ TCEP. The HSET protein was then eluted with 2 × 5 mL 50 mᴍ HEPES pH 8, 0.5 ᴍ NaCl, 5% glycerol, 500 mᴍ imidazole and 2 mᴍ TCEP. The elution fractions were pooled and further purified through an S75 16/60 column, equilibrated with 20 mᴍ Tris pH 7.5, 200 mᴍ NaCl, 2mᴍ TCEP and 5% glycerol, in 5 mL runs.

Colony Forming Unit – Granulocyte Macrophage (CFU – GM) Assay

IMDM media (StemCell Technologies, Grenoble, France) and MethoCult media (H4025 optimum without EPO, StemCell Technologies, Grenoble, France) were thawed overnight at 4 °C. The MethoCult media was then shaken vigorously and allowed to settle before use. The cells used for the assay were prepared as follows. Primary adult human bone marrow mononuclear cells, isolated from the posterior iliac crest (catalogue number ABM007F, StemCell Technologies, Grenoble, France), were thawed at 37 °C and suspended in 13 mL IMEM media. The suspension was centrifuged at 300 g for 10 mins, supernatant was removed, and cells were resuspended. The cell number and viability were then counted. At the same time, compound treatments were prepared as follows. In order to dose in triplicate, 4.4 mL of each compound concentration was prepared in media, with final DMSO concentrations of 0.2%. 4 mL MethoCult media was then added to each falcon tube using a syringe and blunt-end needle. The tubes were then subjected to vortex in order to thoroughly mix the contents, and a 2×10^5 cell / mL suspension was prepared. 0.4 mL of the cell suspension were then transfered to each falcon tube containing the MethoCult media and compound, giving the 1:10 v/v ratio critical for

optimal cell growth. Each tube was subjected to a vortex to mix contents thoroughly and allowed to stand for 5 mins, to allow any bubbles to rise to the top. Using a syringe and a blunt-end needle, 1.1 mL of the final cell suspension was transferred into each well of a 6-well plate. The sample plates were transferred into one 15 cm plate with a 35 mm plate filled with water in the center. The assay plate was stored at 37 °C for 14 days before being scored for colony formation.

Transfection. ON-TARGET plus Human KIFC1 siRNA (catalogue number J-004958-09-005, sequence GGACUCAAACG UUGGACCA) and ON-TARGET plus Mouse Kifc5b siRNA (catalogue number J-047974-08-0005, sequence CCAAG AUGCUUAUGUU UGU) were purchased from Thermo Scientific (Northumberland, UK). A BLAST (alignment) search was conducted using the sequence of ON-TARGET plus Mouse Kifc5b siRNA, against the mouse genome and transcription database, and showed a 100% match to mouse *Kifc1*, and *Kifc5b* genes (both previously encoded *Kifc5a*). This ensured that the correct gene was being knocked-down by the ON-TARGET plus Mouse Kifc5b siRNA. Non-specific Naito 1 Pre-design Chimera RNAi was used as control (catalogue number R0017, Abnova, Heidelberg, Germany). Cells were transfected with Lipofectamine 2000 (catalogue number 11668-019, Invitrogen, Paisley, UK) with a final siRNA concentration of 66 nM. Cells were harvested 24 h, 48 h, and 72 h after transfection and analyzed by Western blotting using an HSET specific antibody.

Western Blotting. Cells were harvested whilst growing in exponential phase by scraping, and lysed in RIPA lysis buffer (50 mM Tris HCl pH 8.0, 150 mM NaCl, 1% NP-

40, 0.5% sodium deoxycholate, 0.1% SDS) and 20 μg, measured using Bradford assay reagent (Biorad, Hemel Hempstead, UK) were separated by SDS-PAGE using Novex Bris-Tris 4-12% gels (Invitrogen, Paisley, UK) in MOPs buffer (Invitrogen, Paisley, UK) and then transferred to nitrocellulose membrane (Invitrogen, Paisley, UK). The membranes were blocked with Odyssey blocking buffer (Li-Cor, Lincoln, USA) for 1 h at rt and then probed with mouse antibodies against HSET (1:5000, KIFC1 (M-63), catalogue number sc-100947, Santa Cruz Biotechnology, Dallas, USA) and anti-β actin antibody (1:5000, catalogue number ab8227, Abcam, Cambridge, UK) overnight at 4 °C. After three washes with Phosphate Buffered Saline (PBS+), 0.1% Tween, the blots were incubated with a goat anti-mouse secondary antibody (1:1000, IRDye 800CW Goat anti-Mouse IgG, catalogue number 926-32210, Li-Cor, Lincoln, USA) for 45 mins at rt. The blots were visualized on Odyssey Infrared Imaging System (Li-Cor, Lincoln, USA). The bands were quantified on the Odyssey Infrared Imaging System (Li-Cor, Lincoln, USA), using ImageQuant TL (GE Healthcare, Buckinghamshire, UK).

Differential Scanning Fluorimetry (DSF). The DFS experiments were carried out using motor domain HSET protein. Reactions were performed in white PCR plates, total volume 20 μL consisting of: 5 μL buffer (HEPES 100 mM pH 7.5), 2 μL NaCl (1.5 M), 2 μL HSET protein (1.6 mg/mL), 4 μL Sypro Orange, 4 μL CW069 solution (20% in DMSO, or 20% DMSO control solution), 3 μL H_2O. The stock CW069 solution was diluted to final assay concentrations of 0.03125 mM, 0.0625 mM, 0.125 mM, 0.25 mM, 0.5 mM and 1 mM in 4% DMSO. The reaction plates were stored at rt for 20 mins to allow the reactions to reach equilibrium before Sypro Orange was added and the experiment run using a Mx3005p Thermal Cycler (Agilient Technologies, Berkshire,

UK). Data was exported into Excel analysis software and a Boltzmann analysis was carried out on the denaturation curves identified by the DSF analysis spreadsheets. The T_m values generated by the Boltzmann analysis were then compared to the mean T_m for HSET motor domain with solvent control, allowing the ΔT_m values for each CW069 concentration to be generated.

SUPPLEMENTARY REFERENCES

Bjelkmar, P., Larsson, P., Cuendet, M.A., Hess, B., and Lindahl, E. (2010). Implementation of the CHARMM Force Field in GROMACS: Analysis of Protein Stability Effects from Correction Maps, Virtual Interaction Sites, and Water Models. J. Chem. Theory Comput. 6, 459-466.

Brooks, B.R., Brooks, C.L., 3rd, Mackerell, A.D., Jr., Nilsson, L., Petrella, R.J., Roux, B., Won, Y., Archontis, G., Bartels, C., Boresch, S., et al. (2009). CHARMM: the biomolecular simulation program. J. Comp. Chem. 30, 1545-1614.

Bussi, G., Donadio, D., and Parrinello, M. (2007). Canonical sampling through velocity rescaling. J. Chem. Phys. 126, 014101.

Essmann, U., Perera, L., Berkowitz, M.L., Darden, T., Lee, H., and Pedersen, L.G. (1995). A Smooth Particle Mesh Ewald Method. J. Chem. Phys. 103, 8577-8593.

Eswar, N., Webb, B., Marti-Renom, M.A., Madhusudhan, M.S., Eramian, D., Shen, M.Y., Pieper, U., and Sali, A. (2006). Comparative protein structure modeling using Modeller. Curr. Protoc. Bioinformatics Chapter 5, Unit 5 6.

Grenga, P.N., Sumbler, B.L., Beland, F., and Priefer, R. (2009). Reductive amination agents: comparison of Na(CN)BH3 and Si-CBH. Tetrahedron Lett. 50, 6658-6660.

Hendlich, M., Bergner, A., Gunther, J., and Klebe, G. (2003). Relibase: design and development of a database for comprehensive analysis of protein-ligand interactions. J. Mol. Biol. 326, 607-620.

Hess, B., Bekker, H., Berendsen, H.J.C., and Fraaije, J.G.E.M. (1997). LINCS: A linear constraint solver for molecular simulations. J. Comp. Chem. 18, 1463-1472.

Hess, B., Kutzner, C., van der Spoel, D., and Lindahl, E. (2008). GROMACS 4: Algorithms for highly efficient, load-balanced, and scalable molecular simulation. J. Chem. Theory Comput. 4, 435-447.

Humphrey, W., Dalke, A., and Schulten, K. (1996). VMD: visual molecular dynamics. J. Mol. Graphics 14, 33-38.

Korb, O., Monecke, P., Hessler, G., Stutzle, T., and Exner, T.E. (2010). pharmACOphore: multiple flexible ligand alignment based on ant colony optimization. J. Chem. Inf. Model. 50, 1669-1681.

MacKerell, A.D., Bashford, D., Bellott, M., Dunbrack, R.L., Evanseck, J.D., Field, M.J., Fischer, S., Gao, J., Guo, H., Ha, S., et al. (1998). All-atom empirical potential for molecular modeling and dynamics studies of proteins. J. Phys. Chem. B. 102, 3586-3616.

Parrinello, M., and Rahman, A. (1981). Polymorphic Transitions in Single-Crystals - a New Molecular-Dynamics Method. J. Appl. Phys. 52, 7182-7190.

Sadowski, J., and Gasteiger, J. (1993). From Atoms and Bonds to 3-Dimensional Atomic Coordinates - Automatic Model Builders. Chem. Rev. 93, 2567-2581.

Vanommeslaeghe, K., Hatcher, E., Acharya, C., Kundu, S., Zhong, S., Shim, J., Darian, E., Guvench, O., Lopes, P., Vorobyov, I., et al. (2010). CHARMM general force field: A

force field for drug-like molecules compatible with the CHARMM all-atom additive biological force fields. J. Comp. Chem. 31, 671-690.
