# Supplementary figures and images for: Design, Synthesis, and Biological Evaluation of an Allosteric Inhibitor of HSET that Targets Cancer Cells with Supernumerary Centrosomes
Source: Chem Biol. 2013 Nov 21;20(11):1399–410. doi: 10.1016/j.chembiol.2013.09.012 (PMC3898838; doi:10.1016/j.chembiol.2013.09.012)

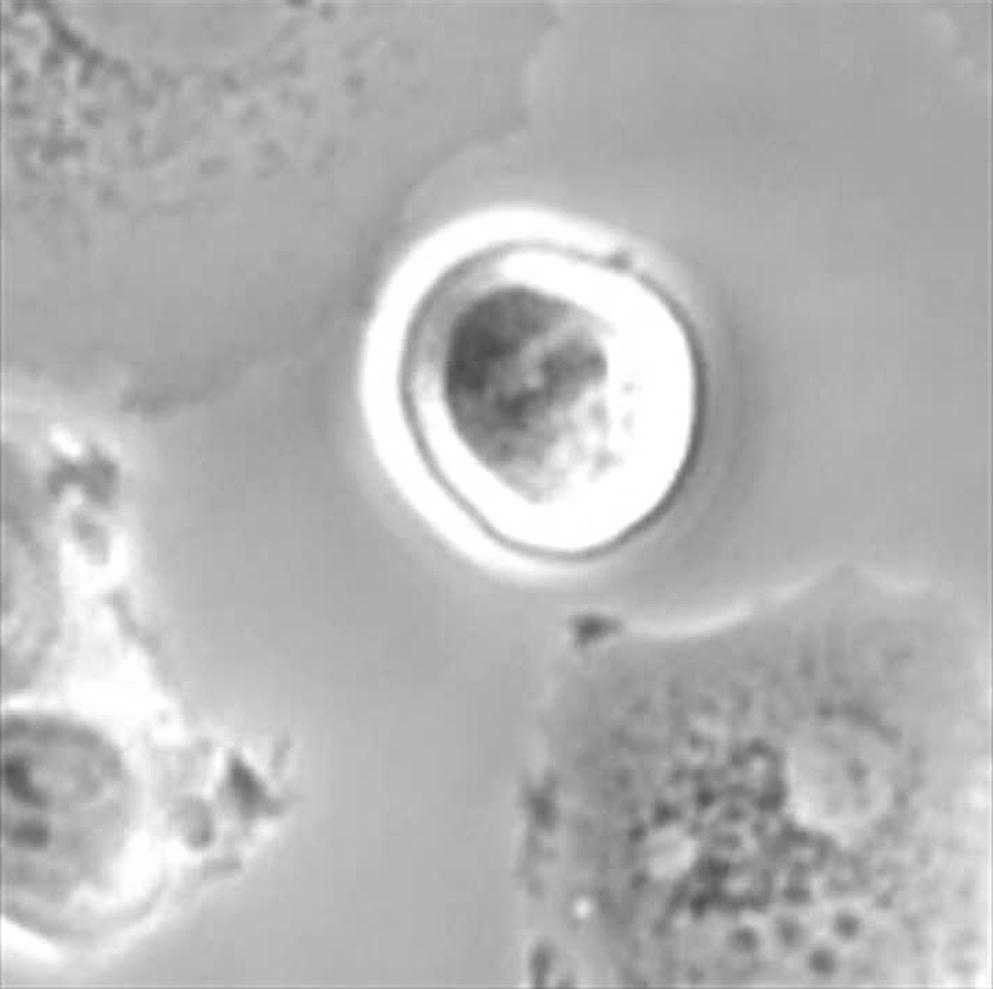

Supplement: Movie S1. Representative Time-Lapse Video of an MCF-7 Cell Treated with DMSO Control Undergoing Bipolar Division [file mmc2.jpg]

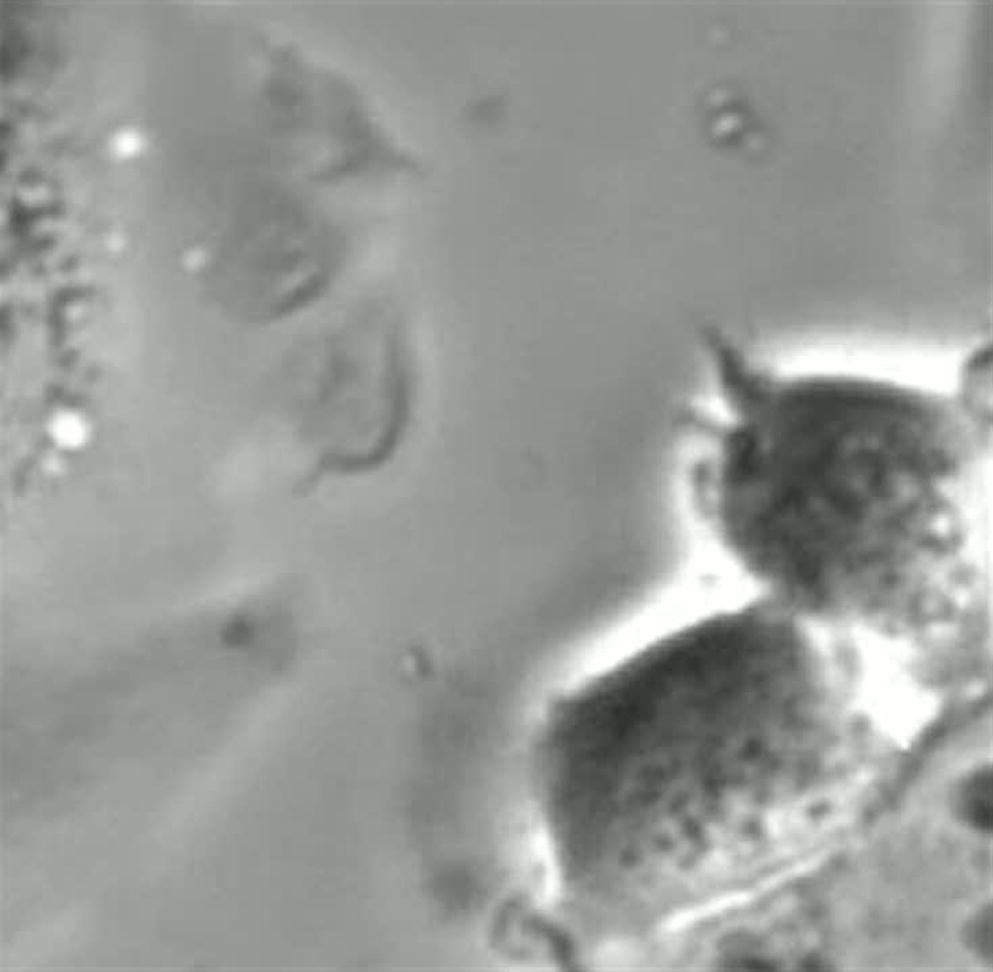

Supplement: Movie S2. Representative Time-Lapse Video of an MCF-7 Cell Treated with 200 μM CW069 Undergoing Bipolar Division [file mmc3.jpg]

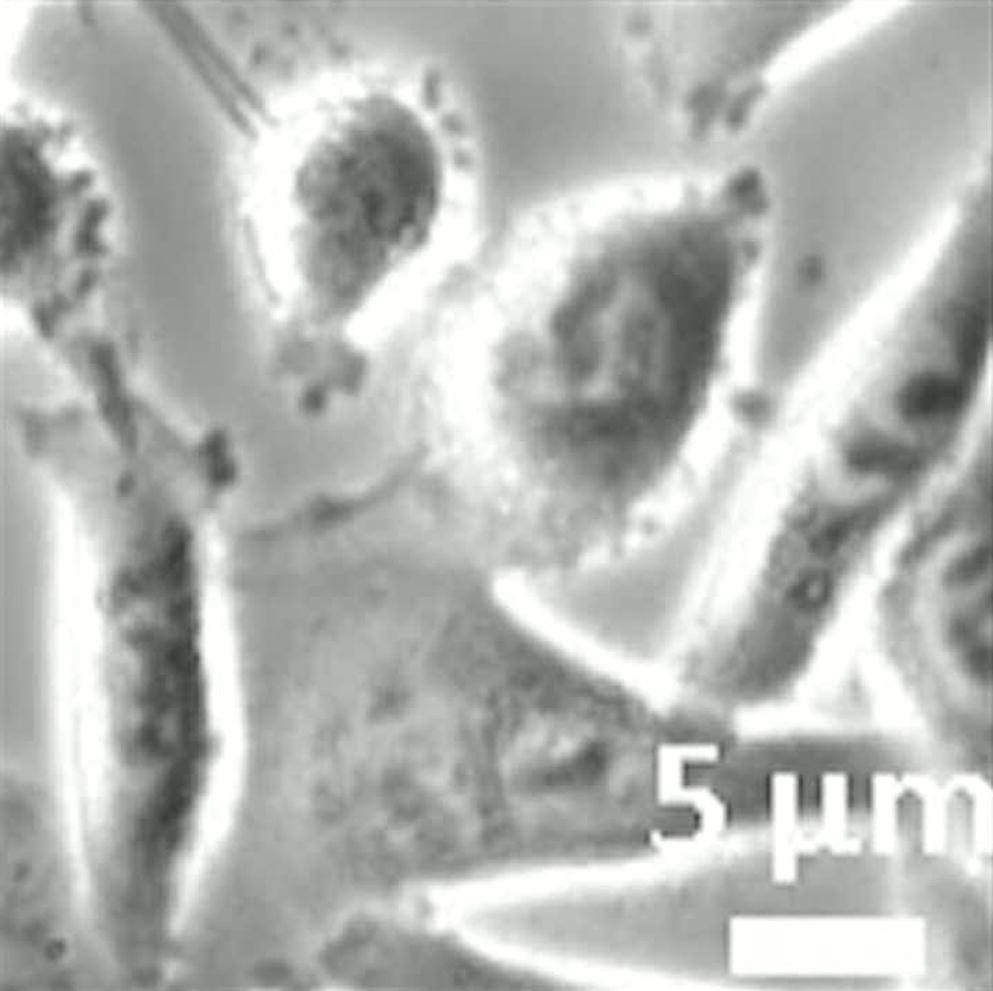

Supplement: Movie S3. Time-Lapse Video Showing HeLa Cells Progressing through Mitosis after 60 Min Treatment with DMSO — Cells were imaged for 360 min. [file mmc4.jpg]

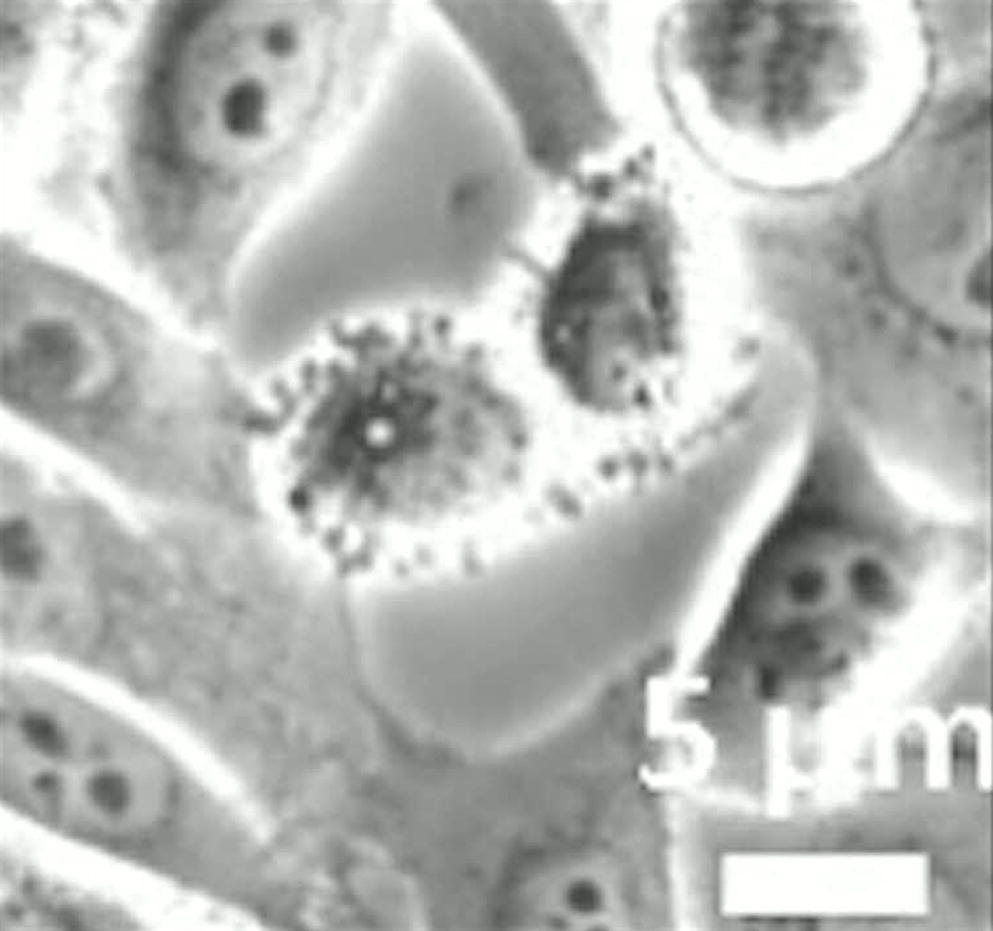

Supplement: Movie S4. Time-Lapse Video Showing HeLa Cells Progressing through Mitosis after 60 Min Treatment with 200 μM CW069 — Cells were imaged for 360 min. [file mmc5.jpg]

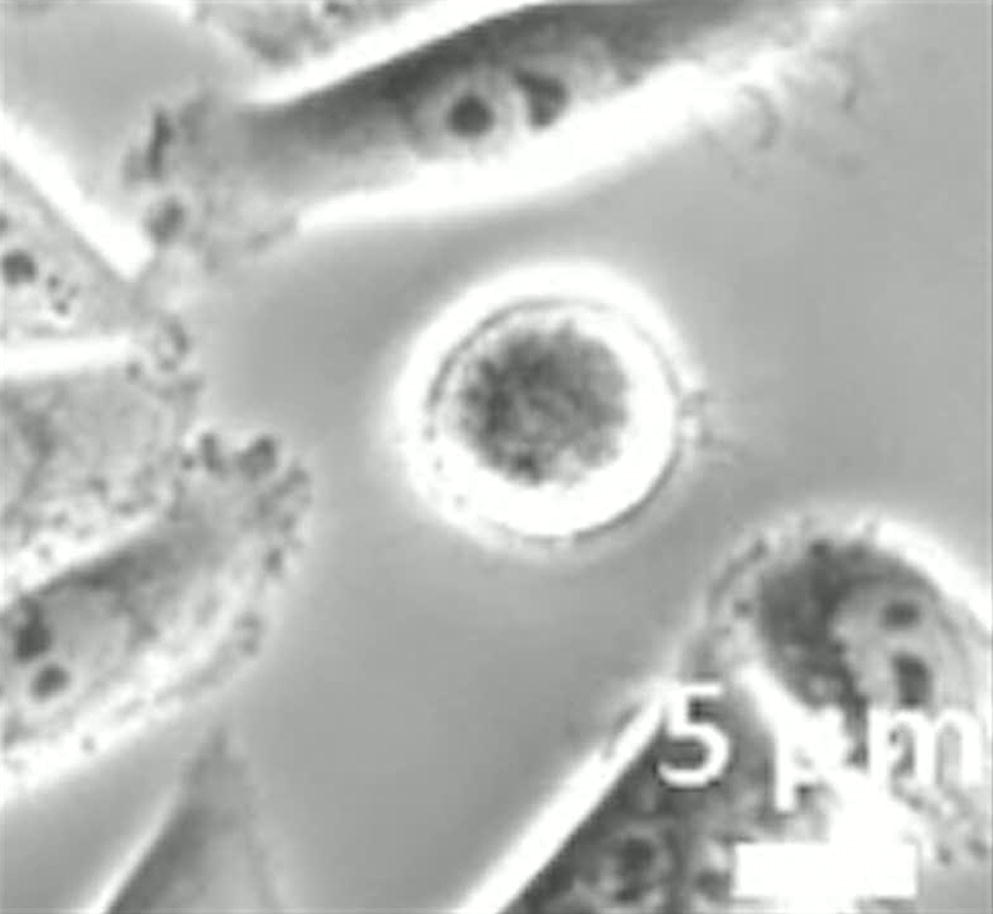

Supplement: Movie S5. Time-Lapse Video Showing HeLa Cells Progressing through Mitosis after 60 Min Treatment with 100 μM Monastrol — Cells were imaged for 360 min. [file mmc6.jpg]

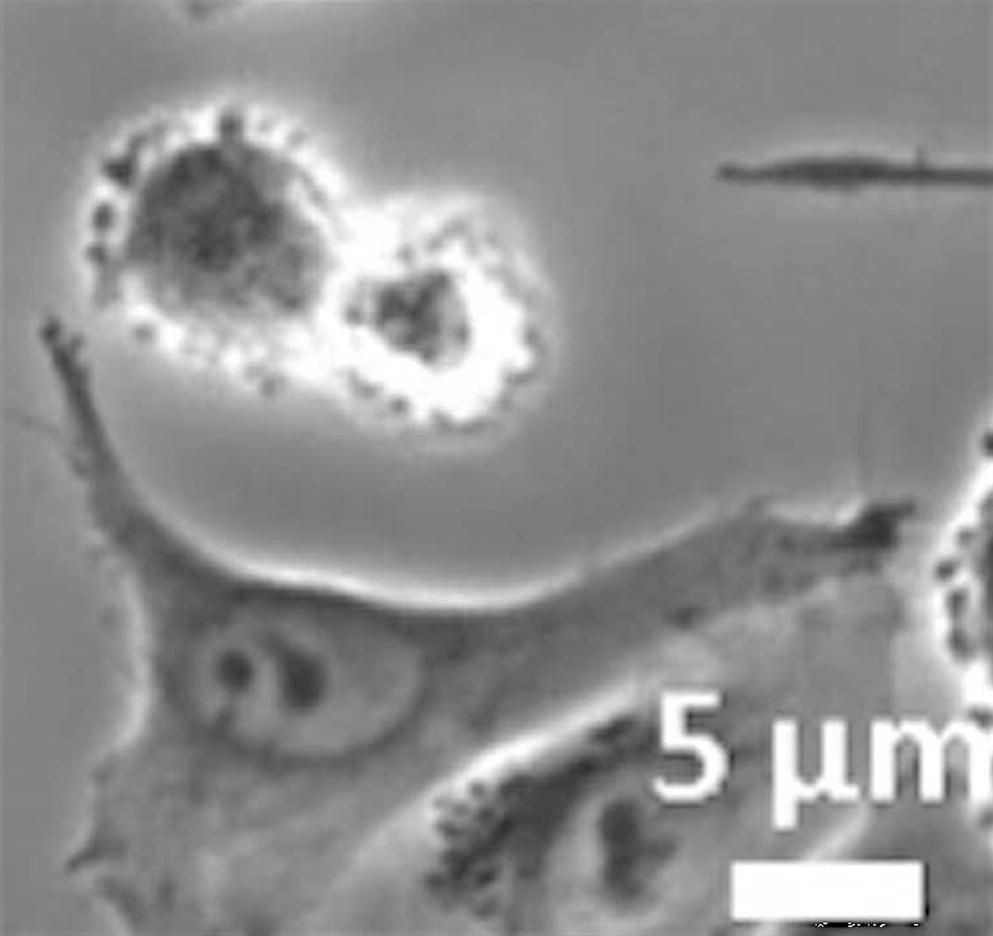

Supplement: Movie S6. Time-Lapse Video Showing HeLa Cells Progressing through Mitosis after 60 Min Treatment with 200 μM CW069 + 100 μM Monastrol — Cells were imaged for 360 min. [file mmc7.jpg]
